# Novel Insights into the Pleiotropic Neuroprotective Action of Synthetic Halogen Free Thyronamine-like Analogues

**DOI:** 10.3390/molecules31162833

**Published:** 2026-08-14

**Authors:** Massimiliano Runfola, Beatrice Polini, Anna Mazzierli, Lorenzo Raffellini, Fabio Di Ricco, Italo Cirone, Simona Sagona, Sheraz Gul, Marco Lessi, Angela Rosa Cuzzola, Rosarita D’Orsi, Fabio Bellina, Clementina Manera, Grazia Chiellini, Simona Rapposelli

**Affiliations:** 1 Department of Pharmacy, University of Pisa, Via Bonanno 6, 56126 Pisa, Italy; massimiliano.runfola@unipd.it (M.R.); lorenzo.raffellini@phd.unipi.it (L.R.); fabio.diricco@phd.unipi.it (F.D.R.); italo.cirone@phd.unipi.it (I.C.); simona.sagona@unipi.it (S.S.); clementina.manera@unipi.it (C.M.); 2 Department of Surgical, Medical, Molecular Pathology and Critical Care, University of Pisa, Via Savi 10, 56100 Pisa, Italy; beatrice.polini@unipi.it (B.P.); anna.mazzierli@phd.unipi.it (A.M.); 3 Fraunhofer Institute for Translational Medicine and Pharmacology ITMP, Discovery Research ScreeningPort, 22525 Hamburg, Germany; sheraz.gul@itmp.fraunhofer.de; 4 Department of Chemistry and Industrial Chemistry, University of Pisa, Via Giuseppe Moruzzi 13, 56124 Pisa, Italy; marco.lessi@unipi.it (M.L.); angela.rosa.cuzzola@unipi.it (A.R.C.); rosarita.dorsi@unipi.it (R.D.); fabio.bellina@unipi.it (F.B.); 5 Center for Instrument Sharing, University of Pisa (CISUP), Lungarno Pacinotti 43, 56126 Pisa, Italy

**Keywords:** diaryl-methane derivatives, thyronamine-like compounds, neuroprotection, anti-inflammatory, *C. elegans*, phenotypic screening

## Abstract

Alzheimer’s disease (AD) is a multifactorial neurodegenerative disorder involving metabolic impairment, neuroinflammation, synaptic failure, and comorbidities. Hence, therapeutic development for AD is rapidly shifting from a single-target approach, centred on amyloid-beta (Aβ) reduction, to multi-target strategies. In this study, we investigated the neuroprotective profile of two acetanilide derivatives, SG-22 and SG-23, originated from the halogen-free thyronamine-like lead compound SG-2. Their efficacy was evaluated through an integrated approach combining in vitro cellular models, in vivo phenotypic screening in a *Caenorhabditis elegans* AD model, and comprehensive ADME-Tox profiling. In U87MG cells, both SG-22 and SG-23 effectively prevented Aβ_25–35_-induced cytotoxicity and restored autophagy-related gene expression, including LC3, SIRT1, and SIRT6, while reducing mTOR and SIRT5 levels. Furthermore, all compounds exhibited anti-inflammatory effects in activated HMC3 microglial cells, reducing IL-6 and increasing IL-10 levels, with evidence suggesting partial involvement of TAAR1 signalling. ADME-Tox analyses revealed improved safety and metabolic profiles for the tested compounds, particularly SG-22, which showed reduced *h*ERG liability and enhanced cytochrome P450 stability. However, in vivo studies demonstrated that only SG-2 and SG-23 improved motility and fitness in the *C. elegans* AD model, consistent with their ability to activate autophagy, whereas SG-22 was ineffective due to limited organismal uptake. Ultimately, the monoacetylated analogue SG-23 emerges as a promising candidate, balancing neuroprotective efficacy and drug-like properties, and supporting thyronamine-like analogues as multi-target agents for AD.

## 1. Introduction

Alzheimer’s disease (AD) is the leading cause of dementia and, consequently, a major contributor to disability among older adults, posing a significant burden on patients, caregivers, and healthcare systems worldwide [1]. By 2050, the global population aged 65 years and older is projected to reach 1.5 billion, and the number of people living with dementia is expected to increase to 139 million worldwide, highlighting the urgent need for effective therapeutic interventions [2].

Despite over a century of research into its etiopathogenesis, AD continues to elude a unifying pathogenic model. Instead, it is increasingly recognized as a complex, multifactorial disorder involving an intricate network of dysregulated pathways, including impaired proteostasis, autophagy dysfunction, microglia-mediated neuroinflammation, and altered lipid metabolism [3,4,5,6]. As a result, AD remains one of the most challenging targets in drug discovery. As of 2025, there are still no therapies capable of effectively halting or even slowing disease progression [7,8].

In this context, it is critical to identify and advance new disease-modifying agents capable of targeting multiple upstream events in AD pathogenesis and restoring systemic—or at least neuronal—homeostasis. To this end, we adopted a collaborative, cross-species approach to investigate the polypharmacological profile of small molecules from our in-house compound library. This strategy led to the identification of SG-2, a synthetic, halogen-free thyronamine-like analogue, as a pleiotropic neuroprotective candidate for AD treatment [9,10,11]. SG-2 exerts pro-cognitive, anti-amnestic, and pro-autophagic effects in rodents, and promotes the recovery of motility and memory in transgenic *Caenorhabditis elegans* and mouse models of AD, respectively [9,12].

Although SG-2 has emerged as a promising candidate for the treatment of neurodegenerative disorders, its in vivo toxicological evaluation in a vertebrate model (zebrafish) revealed a mild but measurable impact on neurodevelopment [11].

**Figure 1:**
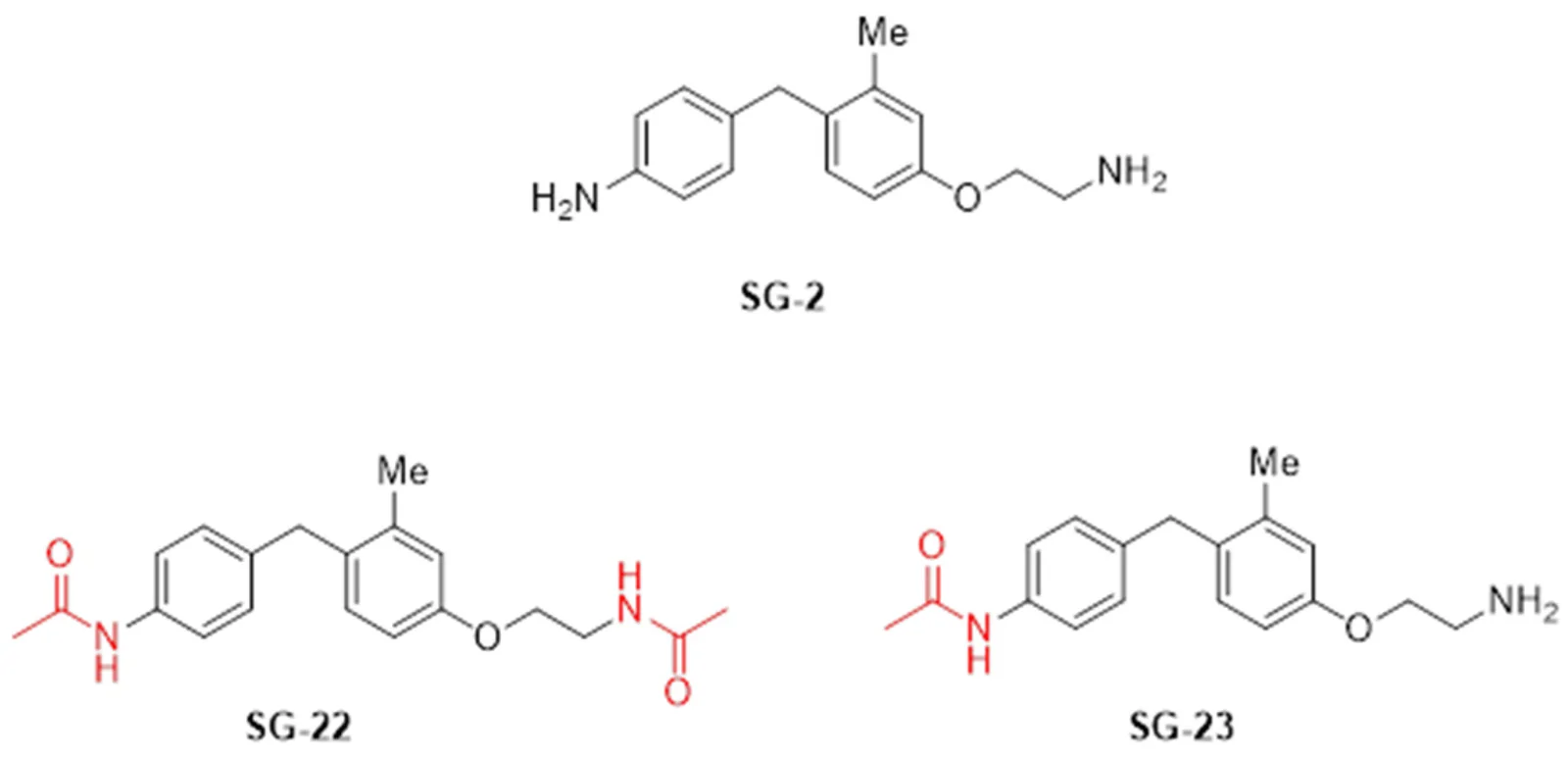
Synthetic thyronamine-like analogues.

In light of this finding, and motivated by the therapeutic potential of the scaffold, we shifted our focus toward previously synthesized acetanilide derivatives of SG-2 [13], namely SG-22 and SG-23 (Figure 1), specifically representing the diacetylated and monoacetylated analogues of SG-2, respectively. We hypothesized that they might retain the neuroprotective properties of the parent compound while exhibiting improved stability and an enhanced safety profile [14,15].

To further investigate the potential neuroprotective effects of these novel drug candidates, we began by exploring their ability to counteract β-amyloid-induced neurotoxicity [16] and microglial inflammation using in vitro models of AD [12,17]. In parallel, we conducted a comparative in vivo assessment of their capacity to promote autophagy and extend lifespan in a *C. elegans* model of AD [18]. Drug-likeness and potential liabilities were also evaluated using an in vitro multiparametric approach [19,20].

## 2. Results

### 2.1. SG-2, SG-22 and SG-23 Prevent Amyloid β_25–35_ Neurotoxicity in U87MG Cells

Based on previous evidence suggesting a potential multi-target neuroprotective activity for the thyronamine-like lead compound SG-2 [11], we investigated whether the two analogues SG-22 and SG-23 [13] were able to maintain the neuroprotective properties of the parent compound.

**Figure 2:**
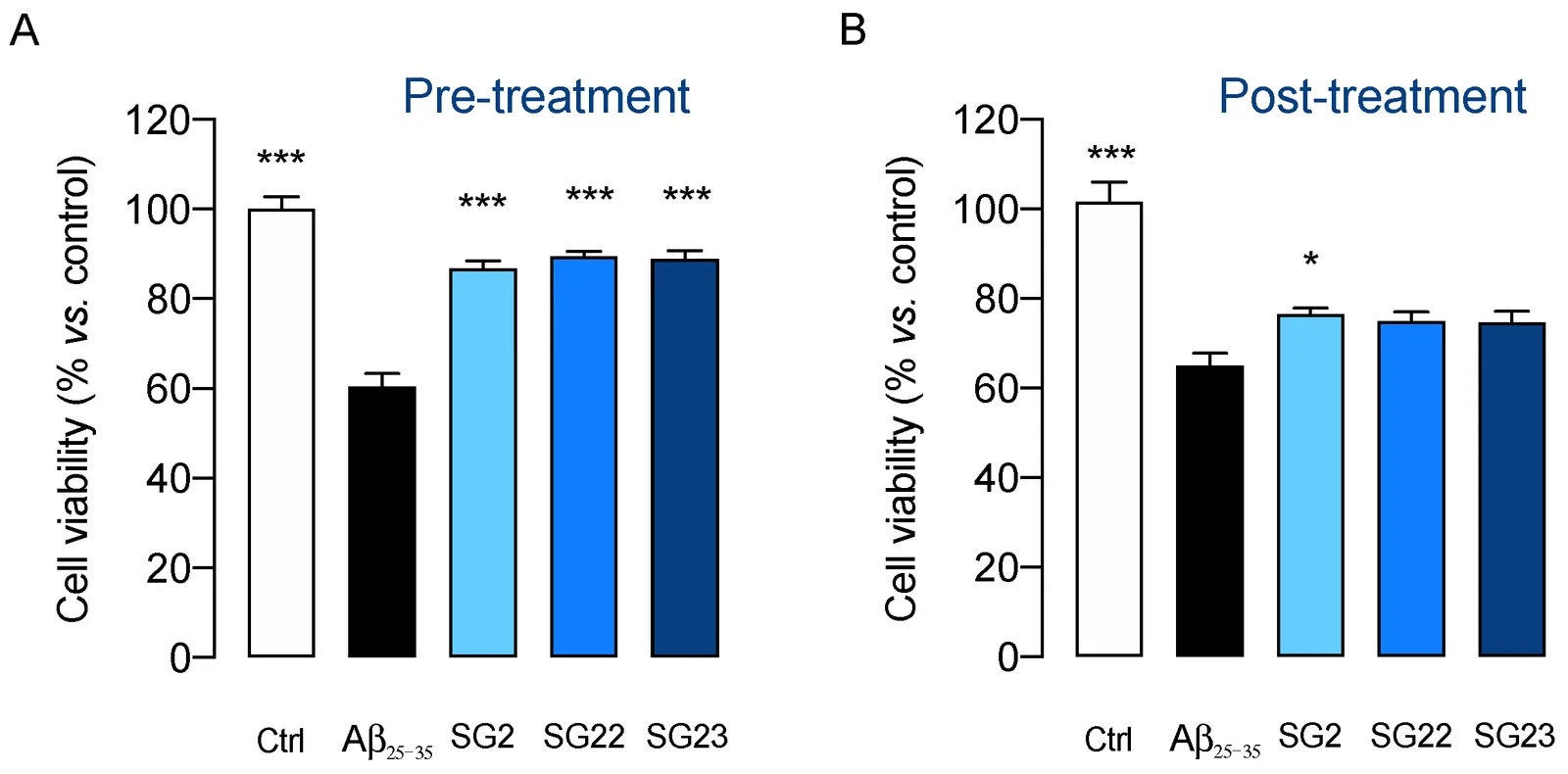
Protective effects of 10 µM SG-2, SG-22, and SG-23 against Aβ_25–35_-induced cytotoxicity in U87MG cells. (**A**) Pre-treatment protocol: U87MG cells were pretreated with 10 μM SG-2, SG-22, or SG-23 for 24 h prior to exposure to Aβ_25–35_ (10 μM; 48 h). (**B**) Post-treatment protocol: cells were first exposed to Aβ_25–35_ (10 μM; 48 h), followed by treatment with SG-2, SG-22, or SG-23 (10 μM, 24 h). Cell viability was assessed as percentage relative to untreated control cells. Data are expressed as mean ± SEM, each performed in technical quadruplicate. Statistical analysis was performed by ordinary one-way ANOVA followed by Tukey’s multiple comparison test. * *p* < 0.05 and *** *p* < 0.005 compared to Aβ_25–35_-exposed cells.

In our experimental setting, exposure of human glioblastoma U87MG cells to 10 μM amyloid β_25–35_ (Aβ_25–35_) for 48 h was found to produce a significant reduction (~40%) in cell viability (Figure 2). Notably, 24 h pretreatment with SG-22 or SG-23, at a concentration of 10 μM, efficiently prevented Aβ_25–35_-induced neurotoxicity, displaying an efficacy comparable to that of parent compound SG-2 (Figure 2A). In contrast, 24 h post-treatment with test compounds at 10 μM dose was nearly ineffective in restoring cell viability in U87MG cells challenged with Aβ_25–35_ (Figure 2B).

**Figure 3:**
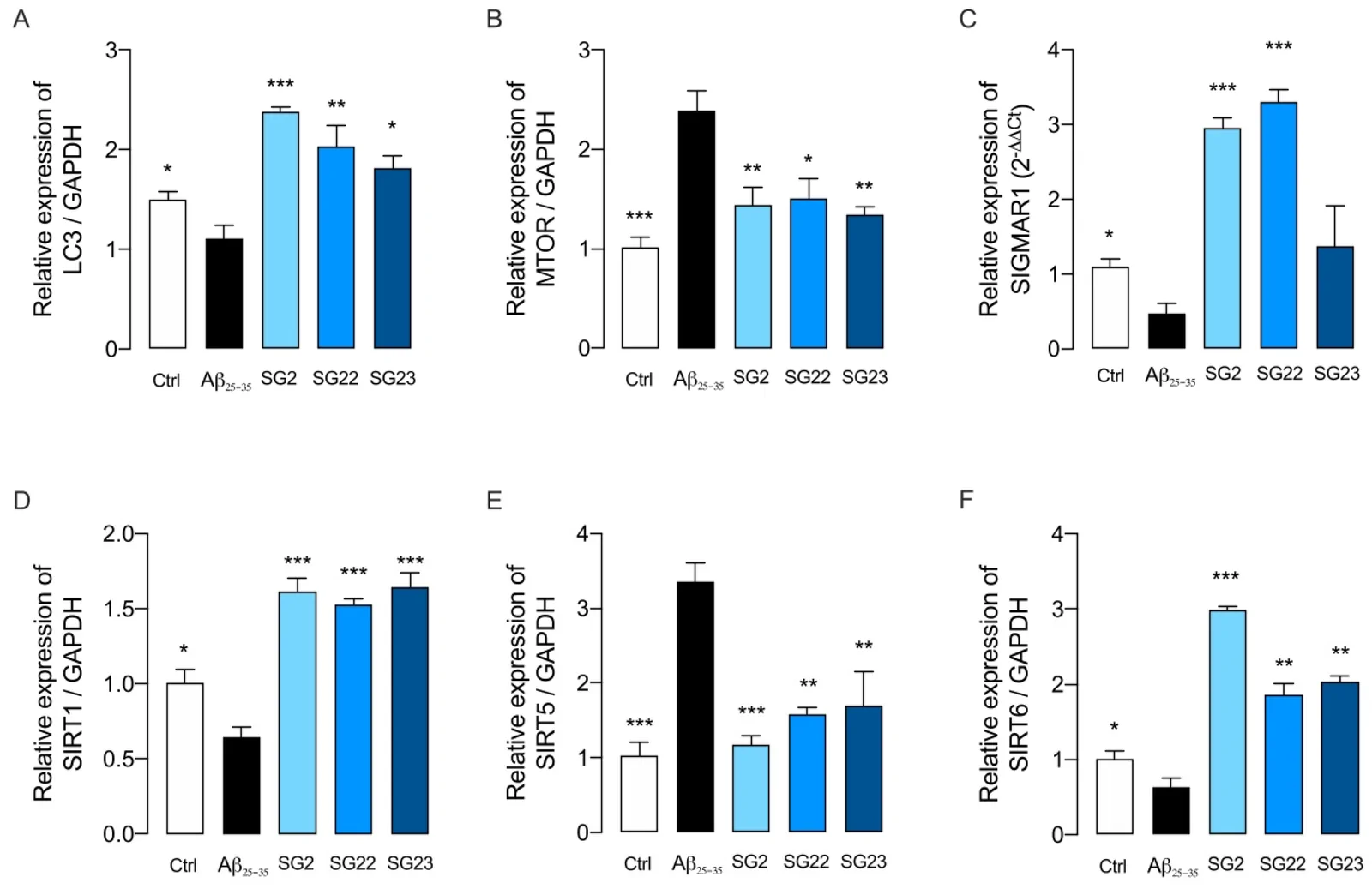
Modulation of autophagy-related gene expression by SG-2, SG-22, and SG-23 in Aβ_25–35_-treated U87MG cells. U87MG cells were pretreated with SG-2, SG-22, or SG-23 (10 μM) prior to exposure to Aβ_25–35_ (10 μM, 48 h). Transcriptional levels of autophagy-related markers, including LC3 (**A**), MTOR (**B**), SIGMAR1 (**C**), SIRT1 (**D**), SIRT5 (**E**), and SIRT6 (**F**), were assessed by qPCR. Data are expressed as fold change relative to vehicle-treated cells, used as control (Ctrl). Data are presented as mean ± SEM, each performed in technical triplicate. Statistical analysis was performed by ordinary one-way ANOVA followed by Tukey’s multiple comparison test. * *p* < 0.05, ** *p* < 0.01 and *** *p* < 0.005 compared to Aβ_25–35_-exposed cells.

There is substantial evidence that autophagy flux (ATG) is impaired in AD [3,21]. Therefore, we investigated whether pretreatment with the compounds under examination could prevent the deleterious transcriptional effects on the autophagic pathway induced by exposure of U87MG cells to Aβ_25–35_ (Figure 3). Consistent with previous evidence [11], pretreatment with SG-2, SG-22, or SG-23 (10 μM) was able to prevent the transcriptional impairment of autophagic flux induced by Aβ_25–35_ (Figure 3), restoring the expression of key pro-autophagic genes, such as LC3 (microtubule-associated protein 1 light chain 3), SIRT1 (sirtuin 1) and SIRT6 (sirtuin 6) [22,23,24] to levels comparable or even higher than those observed in control U87MG cells. In parallel, these compounds significantly counteracted the Aβ-induced upregulation of negative regulators of autophagy, such as mTOR (mammalian target of rapamycin), and SIRT5 (sirtuin 5) [25,26].

Taken together, our data indicate that analogues SG-22 and SG-23 are capable of preventing β-amyloid induced deleterious transcriptional effects on ATG, in a manner similar to that demonstrated by the lead compound SG-2. These findings suggest that the two thyronamine-like analogues may hold promise for the treatment of AD.

### 2.2. Anti-Inflammatory Properties of Thyronamine-like Compounds SG-2, SG-22 and SG-23 in Human Microglial Cells (HMC3)

Considering the key role of microglial activation in the onset and progression of several neurodegenerative disorders [27], we investigated the effects of thyronamine-like analogues SG-2, SG-22 and SG-23 against LPS/TNFα-induced neuroinflammation in the HMC3 cell line.

HMC3 cells were pretreated with increasing concentrations (0.1, 1 and 10 μM) of the test compounds for 1 h, followed by stimulation with LPS (10 μg/mL) and TNFα (50 ng/mL) for 24 h. Culture media were then collected, and the concentrations of pro-inflammatory (IL-6) and anti-inflammatory (IL-10) cytokines were determined using specific ELISA assays.

**Figure 4:**
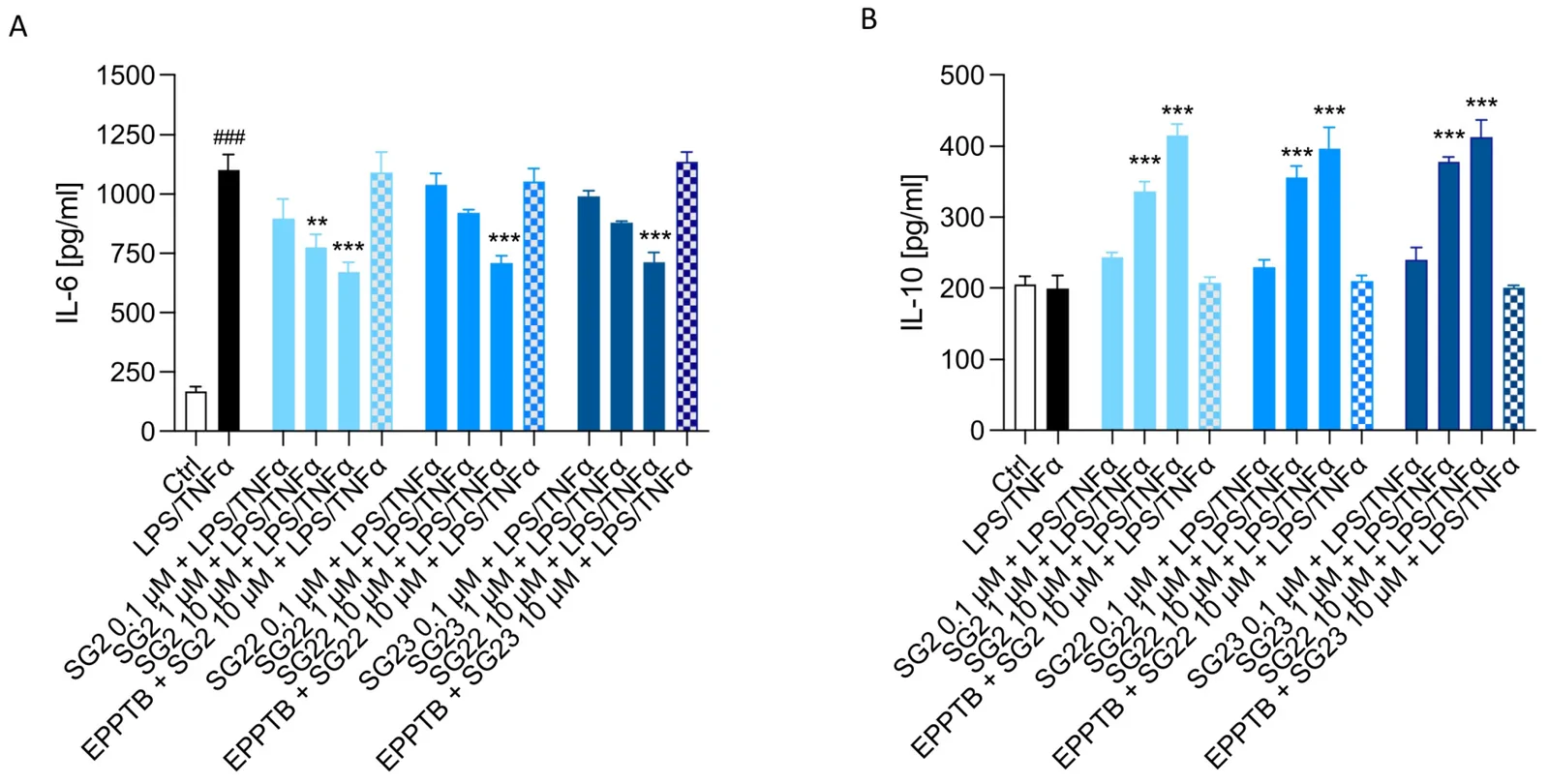
Release of pro-inflammatory IL-6 (**A**) and anti-inflammatory IL-10 (**B**) interleukins in HMC3 cells exposed to LPS/TNFα and treated with SG2, SG22 and SG23 (10 µM; 24 h) in the presence or absence of the TAAR1 antagonist EPPTB (1 μM; 24 h). HMC3 cells were pretreated with increasing concentrations (0.1, 1, and 10 μM) of SG-2, SG-22, or SG-23 for 1 h, in the presence or absence of the selective trace amine-associated receptor 1 (TAAR1) antagonist EPPTB, followed by stimulation with LPS (10 μg/mL) and TNFα (50 ng/mL) for 24 h. Levels of pro-inflammatory (IL-6) and anti-inflammatory (IL-10) cytokines in the culture media were quantified by ELISA. Data are expressed as mean ± SEM, each performed in technical triplicate. Statistical analysis was performed by ordinary one-way ANOVA followed by Tukey’s multiple comparison test. ^###^ *p* < 0.005 ** compared to vehicle-treated cells used as control (Ctrl). ** *p* < 0.01 and *** *p* < 0.005 compared to cells treated with LPS and TNFα.

As shown in Figure 4, the thyronamine-like agents exhibited a significant dose-dependent anti-inflammatory effect in LPS/TNFα-stimulated HMC3, as evidenced by a reduction in pro-inflammatory IL-6 levels and a concomitant increase in IL-10 levels.

Notably, pretreatment with the selective trace-amine associated receptor type 1 (TAAR1) antagonist EPPTB (1 μM) [28] markedly attenuated the anti-inflammatory effects produced by SG-2, SG-22, and SG-23 when used at the highest concentration (10 μM), suggesting a possible involvement of TAAR1 signalling in mediating the anti-inflammatory activity of these novel thyronamine-like analogues. Although still at a preliminary stage, these results appear consistent with the reported ability of thyronamine-like compounds to modulate cellular stress and metabolic responses, which are closely linked to inflammatory signalling [9,12,17,29].

### 2.3. In Vitro Preclinical Safety Profile of Thyronamine-like Analogue SG-2, SG-22 and SG-23

**Figure 5:**
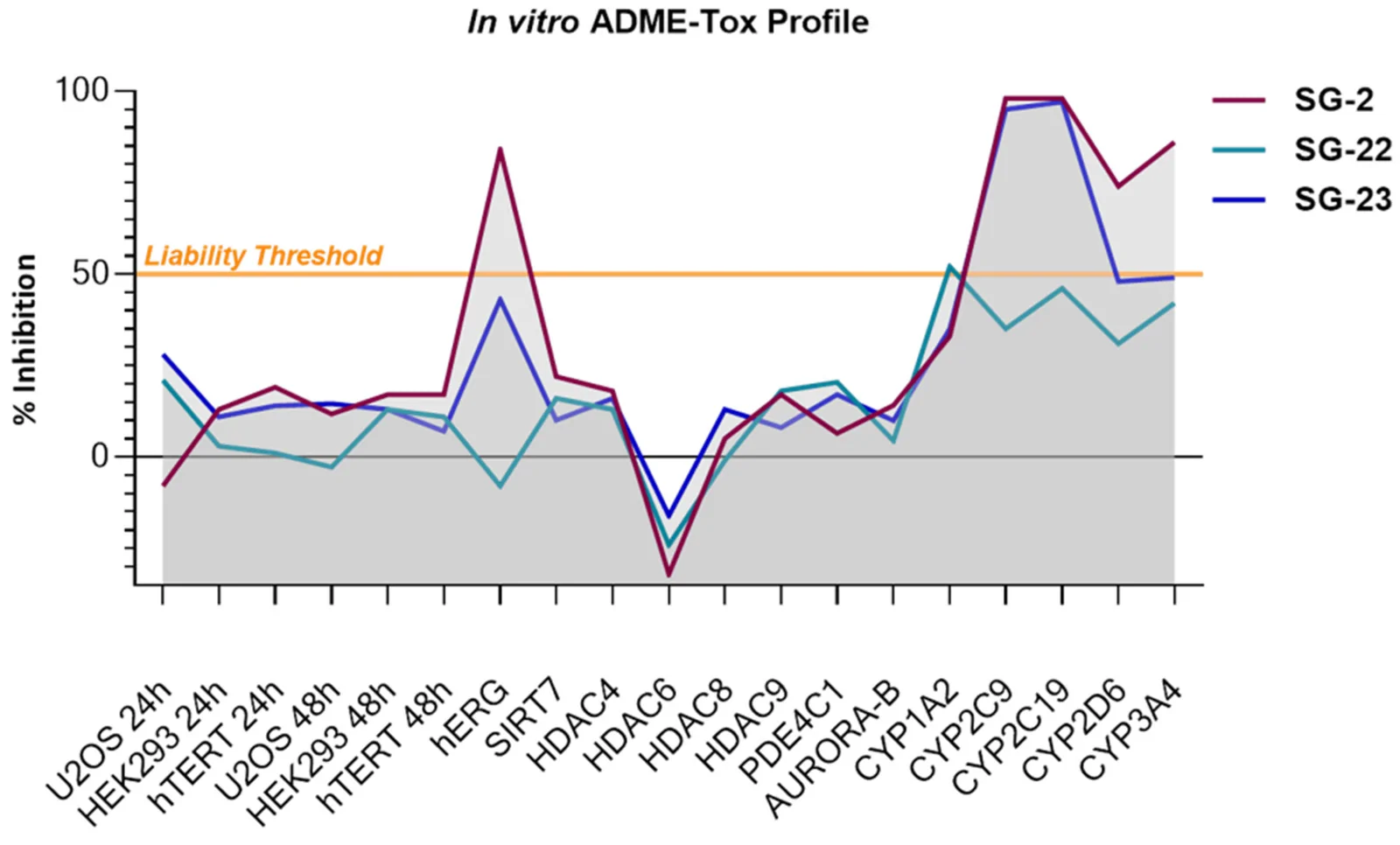
In vitro ADME-Tox profiling of SG-2, SG-22, and SG-23 at 10 μM. Percentage of inhibition measured across a panel of safety-relevant assays, including cytotoxicity (U2OS, HEK293, hTERT at 24 h and 48 h), ion channel interaction (*h*ERG), epigenetic and signalling enzymes (SIRT7, HDAC4, HDAC6, HDAC8, HDAC9), key off-targets (PDE4C1, AURORA-B), and metabolic stability (CYP1A2, CYP2C9, CYP2C19, CYP2D6, CYP3A4). The orange line indicates the predefined liability threshold (50% inhibition). The number of replicates ranged from 2 to 6, and detailed experimental information is provided in the Supplementary Material.

Given the promising pleiotropic neuroprotective activity displayed by the lead thyronamine-like compound SG-2 and its acetanilide derivatives SG-22 and SG-23, we advanced the three compounds into an in vitro preclinical safety profiling campaign to assess drug-likeness and potential liabilities [20]. To this end, we employed a comprehensive panel of assays, including: (i) cytotoxicity evaluation across three representative human cell lines (U2OS, hTERT, and HEK293) to assess general cellular tolerability; (ii) hERG channel inhibition assay to evaluate potential cardiotoxic risk; (iii) off-target profiling encompassing key enzymes and receptors frequently implicated in adverse drug reactions, such as histone deacetylases (HDACs), phosphodiesterase 4C1 (PDE4C1), and Aurora kinase B (AURK B); (iv) cytochrome P450 inhibition panel, covering the five major human isoforms (CYP1A2, CYP2C9, CYP2C19, CYP2D6, and CYP3A4), to predict metabolic interactions and potential for drug–drug interactions. This multi-parametric approach provided a robust initial safety snapshot of SG-2, SG-22 and SG23. As shown in Figure 5, the lead compound SG-2 exhibited marked inhibition of the hERG channel (>85%), raising significant concerns regarding potential cardiotoxicity [30]. In contrast, both analogues, SG-22 and SG-23, demonstrated a more favourable safety profile in this assay, showing substantially lower hERG inhibition (Figure 5), which was particularly evident for the diacetylated analogue SG-22.

In addition to improved hERG liability, the analogues SG-22 and SG-23 demonstrated increased metabolic stability compared to SG-2, likely due to the partial (e.g., SG-23) or full (e.g., SG-22) protection of the amino groups as acetamides, as these groups are particularly susceptible to oxidative metabolism by CYP450 enzymes [31]. Indeed, the analogue SG-2, characterized by the presence of two free amino groups, showed inhibition values above the liability threshold across all CYP isoforms, whereas its diacetylated analogue SG-22 consistently remained below the threshold, indicating a markedly improved metabolic profile. The monoacetylated analogue SG-23, bearing a free alkyl amino group, displayed partial metabolic stability, with reduced inhibition observed for only two CYP isoforms, namely CYP2D6 and CYP3A4, the latter being involved in the metabolism of approximately 50% of clinically used drugs [32].

Notably, all compounds displayed a favourable toxicological profile, causing minimal cytotoxicity across the three representative human cell lines (U2OS, hTERT, and HEK293), and were associated with a significantly reduced off-target liability.

### 2.4. SG-2, SG-23 but Not SG-22 Restores Whole-Population Worm Health in a C. elegans Model of AD via Autophagy Activation

Given the retention of neuroprotective activity and the improved safety and metabolic profiles observed for the SG-2 acetylated analogues SG-22 and SG-23, we decided to assess whether these novel drug candidates could also exert beneficial effects in in vivo models of AD. To this end, we employed the same transgenic *C. elegans* model of AD, which constitutively expresses human Aβ_1–42_ in body wall muscle cells, previously used by us to evaluate SG-2’s ability to slow AD progression [11]. SG-2 was therefore used as a positive control in these experiments. We treated L4-synchronized *C. elegans* worms with 10 μM of SG-2, SG-22, or SG-23. After 144 h (day 6) of drug exposure, we analyzed their behavioural parameters, including motility, force, speed, and moving ratio [33,34], to obtain a comprehensive multiparametric fingerprint of whole-worm population health, referred to as the Total Fitness Score (TFS).

**Figure 6:**
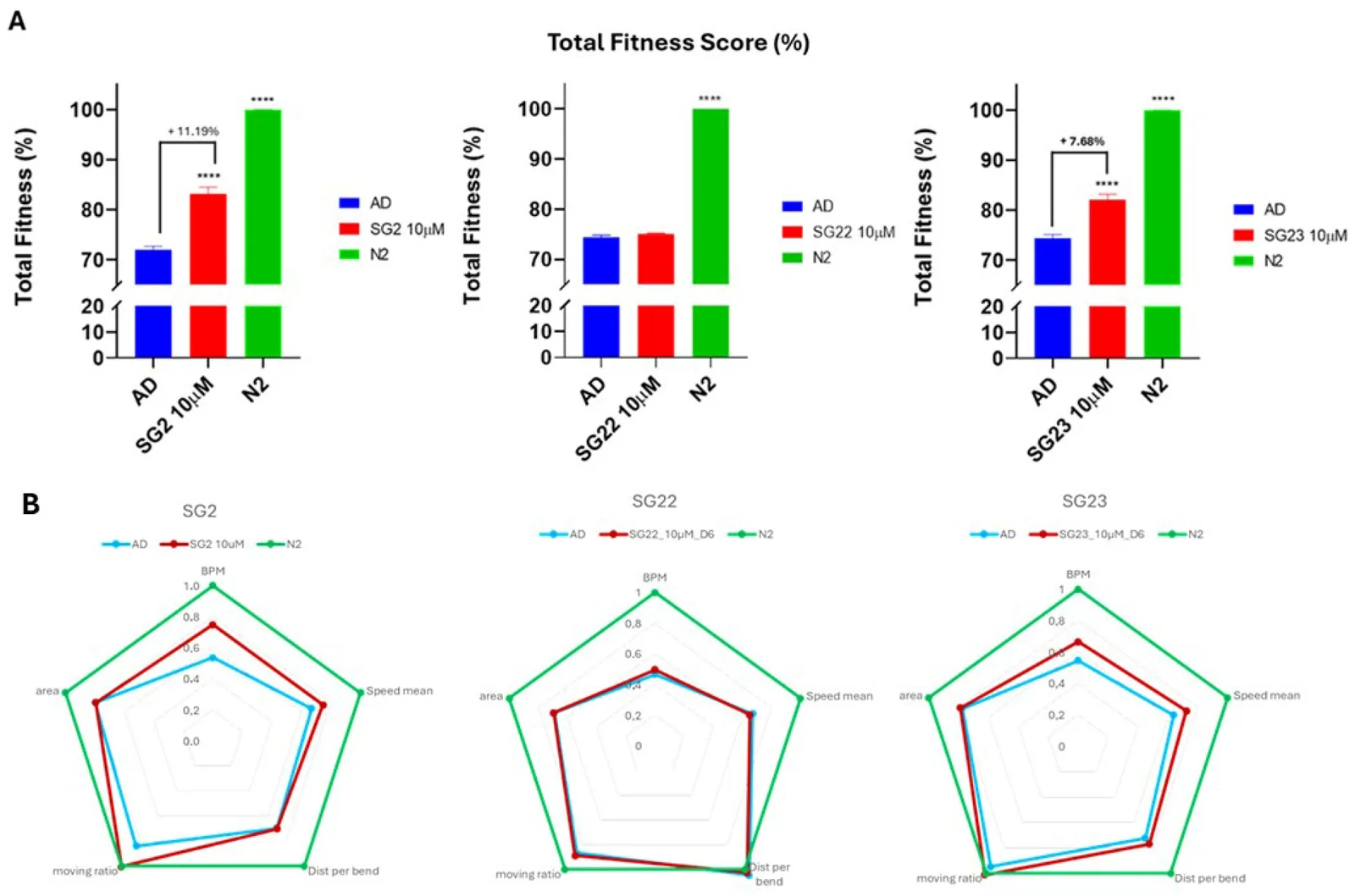
SG2 and SG23, but not SG22 recover AD phenotype in a *C. elegans* model of AD model. (**A**) Bar graph representing the Total Fitness Score of worms treated with SG-2, SG-22, or SG-23. Error bars represent the standard error of the mean (SEM). For statistical tests, one-way ANOVA was used. *p* ≤ 0.0001 (****). (**B**) D6 multi-parameter analysis used to calculate Total Fitness, integrating the following parameters: motility (derived from body bends per minute (BPM)), force (estimated as distance per bend), speed, worm size (based on body area), and motile fraction (percentage of moving animals). Red: AD worms treated with compounds; green: wild-type (N2) controls; blue: untreated AD controls. Experiments were performed in three independent biological replicates, each derived from a separate synchronization batch.

As shown in Figure 6, SG-2 demonstrated the ability to restore the AD phenotype, strongly ameliorating the TFS compared with vehicle-only–treated worms. Similarly, SG-23 induced a partial yet consistent recovery of the AD phenotype. In contrast, SG-22–treated worms showed no improvement, displaying a phenotype indistinguishable from untreated AD controls.

In parallel, to gain mechanistic insight, we performed quantitative PCR analyses to assess whether the autophagy-inducing effect previously observed for SG-2 in *C. elegans* was retained in its analogues. To this end, we evaluated the expression levels of key autophagy-related orthologous genes in *C. elegans*, including beclin 1 (*bec-1*), microtubule-associated protein 1 light chain 3 (*lgg-1*), mechanistic target of rapamycin (*let-363*), sequestosome 1 (*sqst-1*), autophagy-related protein 5 (*atg-5*), and sirtuin 2.1 (*sir-2.1*) [35,36].

**Figure 7:**
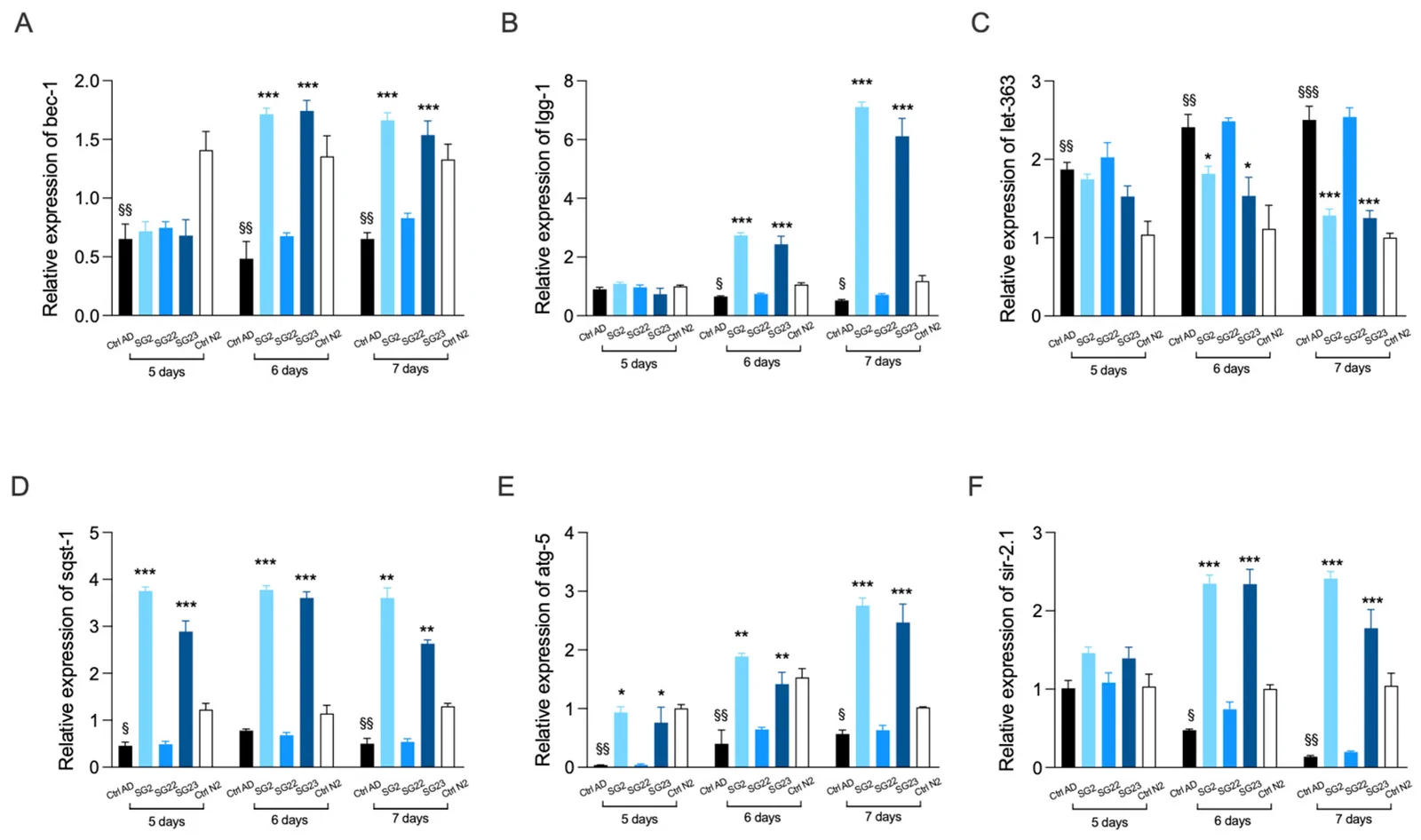
Modulation of autophagy-related gene expression by SG-2, SG-22, and SG-23 in a *C. elegans* model of AD. L4-synchronized transgenic worms expressing human Aβ_1–42_ were treated with SG-2, SG-22, or SG-23 (10 μM) and collected at day 6 (D6). Transcriptional levels of autophagy-related genes, including *bec-1* (**A**), *lgg-1* (**B**), *let-363* (**C**), *sqst-1* (**D**), *atg-5* (**E**), and *sir-2.1* (**F**), were quantified by qPCR. Data are expressed as fold change relative to vehicle-treated AD worms and presented as mean ± SEM. Statistical analysis was performed by ordinary one-way ANOVA followed by Tukey’s multiple comparison test. ^§^ *p* < 0.05, ^§§^ *p* < 0.01 and ^§§§^ *p* < 0.005 compared to wild-type (N2) controls * *p* < 0.05 ** *p* < 0.01 and *** *p* < 0.005 compared to vehicle-treated AD worms. SG-2 and SG-23 significantly modulated the expression of autophagy-related genes, whereas SG-22 showed no significant effect.

As shown in Figure 7, when tested at a concentration of 10 μM, both SG-2 and SG-23 promoted autophagy with comparable potency.

Unfortunately, worms treated with SG-22 showed no changes in the expression of autophagy-related biomarkers, consistent with the impaired motility phenotype previously observed. Overall, the PCR analysis suggests, on the one hand, that SG-22 may not be efficiently absorbed by nematodes and, on the other hand, that the neuroprotective effects observed in these organisms are closely linked to activation of the autophagic process.

Regarding the analogues SG-2 and SG-23, the present data confirm that the pro- autophagic activity detected in vitro effectively translates into a positive neuroprotective response in vivo. In contrast, the lack of activity observed for SG-22, together with the absence of changes in autophagy-related gene expression in *C. elegans*, may be explained, at least in part, by the increased lipophilicity of the compound, which might have impaired its permeability in *C. elegans*. To explore this hypothesis in more detail, we conducted a quantitative analysis of SG-2, SG-22, and SG-23 uptake in nematodes, as described below.

### 2.5. Compound Uptake Quantification on C. elegans

To evaluate the uptake of SG compounds by *C. elegans*, we performed qualitative and quantitative analyses using Ultra-High-Performance Liquid Chromatography coupled with High-Resolution Mass Spectrometry (UHPLC–HR MS). Analysis of nematode extracts revealed compound concentrations of 7.2 ng/mL (SG-2), 2.7 ng/mL (SG-22), and 10.6 ng/mL (SG-23). The significantly lower concentration detected for SG-22 (approximately 2.5- to 4-fold lower than SG-2 and SG-23, respectively) suggests poor absorption of this compound by the nematodes (Table S1, Supplementary Materials).

Interestingly, despite its minimal uptake, SG-22 was found to be partially metabolized into SG-23 in the worms. Furthermore, UHPLC–HR MS analysis of SG-22 samples revealed the presence of three additional unknown peaks, each with mass-to-charge ratios differing by a single methyl group (*m*/*z* 328, 342, and 356), suggesting the formation of putative metabolites whose structures remain to be elucidated.

To further confirm the limited permeability of SG-22, we quantified the residual compound in the culture medium by an ultra-performance liquid chromatography method with diode array detection (UPLC-MS DAD). Standard solutions of the analyte were prepared to generate a calibration curve using acetanilide as an internal standard (Figure S4). The calibration curve was used to determine the analyte concentration in both the dosage solution (i.e., blank sample solution) and the solution collected after worm treatment (i.e., treatment sample solution).

The blank sample solution consisted of a preparation in which the worm culture medium was used as the solvent, and the compound of interest was dissolved and subsequently analyzed either by direct extraction or after lyophilization, in order to maximize compound recovery.

After optimizing the conditions for compound recovery from the blank sample solution (Table S3), we analyzed the treatment sample solutions. Direct extraction of the blank sample solution with ethyl acetate (AcOEt) proved to be the most reliable method for quantifying SG-22 in the worm environment. The same procedure was applied to the treatment sample solutions, yielding a recovery of 90.9 ± 0.7%. This value, comparable to the recovery obtained from the blank sample solution (91.7% ± 0.7%), confirms the inability of the worms to effectively absorb the compound.

Taken together, the MS data provide clear support for the hypothesis formulated on the loss of biological activity of SG-22 in *C. elegans* due to a reduced uptake of the compound.

## 3. Discussion

Our study provides new evidence supporting the therapeutic potential of synthetic thyronamine-like analogues as multi-target neuroprotective agents for Alzheimer’s disease (AD). By integrating in vitro and in vivo approaches, we demonstrate that structural modification of the lead compound SG-2 yields derivatives with preserved biological activity while improving key drug-like properties, although with important differences in organismal efficacy.

A key finding of this work is that both SG-22 and SG-23 retain the neuroprotective profile of SG-2 in cellular models. In U87MG cells, all compounds effectively prevented Aβ_25–35_-induced cytotoxicity when administered as a pretreatment, highlighting their ability to interfere with early pathogenic events triggered by amyloid toxicity. The lack of efficacy observed in post-treatment conditions additionally implies that these compounds act primarily as preventive modulators of cellular stress responses, rather than as rescuing agents once the pathological effects are already established. This distinction is particularly relevant in AD, where early intervention targeting upstream pathways is increasingly recognized as critical for disease modification [37].

Mechanistically, our data indicate that modulation of autophagy represents a key component of the observed neuroprotective effects. SG-2 and its analogues restored the transcriptional balance of autophagy-related genes disrupted by Aβ exposure, including upregulation of pro-autophagic markers (LC3, SIRT1, SIRT6) and reduction of negative regulators such as mTOR and SIRT5 [38]. These findings are consistent with the well-established role of impaired autophagy in AD pathogenesis and support the hypothesis that restoration of proteostatic mechanisms is a viable therapeutic strategy [39]. Importantly, the ability of SG-22 and SG-23 to reproduce these effects confirms that acetylation of amino groups does not compromise, at least in vitro, the key signalling pathways underlying SG-2 activity.

In parallel, the anti-inflammatory effects observed in human microglial HMC3 cells further extend the pleiotropic profile of these compounds. All three molecules reduced IL-6 release while increasing IL-10 levels under inflammatory stimulation, indicating a shift toward an anti-inflammatory phenotype. The attenuation of these effects by the TAAR1 antagonist EPPTB suggests that TAAR1 signalling may play a significant role in mediating the immunomodulatory activity of thyronamine-like compounds. This is in line with previous evidence linking TAAR1 activation to regulation of cellular metabolism and inflammatory responses [17], and supports the idea that these compounds act through coordinated modulation of neuroinflammation and cellular homeostasis.

A crucial advancement of this study lies in the pharmacokinetic and safety optimization achieved through the SG2-acetylation. The bis-acetylated analogue SG-22, in particular, showed a markedly improved ADME-Tox profile compared to SG-2, including reduced hERG inhibition and enhanced metabolic stability. These findings confirm that masking the basic amino groups is an effective strategy to mitigate cardiotoxic liabilities and improve drug-likeness [40]. However, this chemical optimization revealed an important trade-off between safety and biological efficacy that became evident in the in vivo model.

Indeed, phenotypic screening in the *C. elegans* AD model uncovered a divergence between in vitro and organismal activity. While SG-2 and SG-23 significantly improved worm motility and overall fitness, SG-22 failed to produce any detectable beneficial effect. This discrepancy was mechanistically supported by gene expression data, showing that SG-22 did not activate autophagy in vivo, and by uptake studies demonstrating its markedly reduced bioavailability in worms. The increased lipophilicity associated with double acetylation is likely to impair compound absorption and distribution within the nematode, resulting in inadequate intracellular concentrations to exert its biological activity. These findings highlight that, although *C. elegans* represent a valuable platform for rapid and cost-effective whole-organism screening, its distinct physiology, including compound uptake and metabolism, can influence drug activity. Consequently, negative results may not necessarily reflect a lack of intrinsic efficacy but rather insufficient target exposure. In particular, cuticular permeability may bias the evaluation of highly lipophilic compounds, potentially leading to false-negative outcomes [41,42]. Therefore, validation in mammalian in vivo models is essential to confirm the translational relevance of these findings.

Overall, our results identify SG-23 as a promising compromise between efficacy and drug-like properties. Unlike SG-22, the monoacetylated analogue preserves sufficient polarity to ensure uptake while still benefiting from partial metabolic protection, resulting in retained neuroprotective efficacy in both cellular and organismal models.

Future studies will focus on optimizing the balance between lipophilicity and permeability in SG-2 derivatives, as well as exploring alternative strategies to enhance brain delivery while maintaining safety. In addition, deeper mechanistic studies dissecting the interplay between TAAR1 signalling, autophagy, and neuroinflammation will further clarify the therapeutic potential of this novel class of thyronamine-like compounds.

## 4. Materials and Methods

### 4.1. Compounds

SG2, SG-22 and SG-23 were synthesized as previously reported [13]. The synthetic pathway is depicted in Supplementary Materials. 1H-NMR, 13C-NMR spectra were obtained with a Bruker (Billerica, MA, USA) Avance III 400 MHz spectrometer and were recorded at 400 and 101 MHz, respectively. 13C-NMR spectra are 1H decoupled. Signal spectra were fully decoupled. 1H NMR and 13C NMR spectra are reported in Supplementary Materials.

### 4.2. In Vitro Experiments

#### 4.2.1. Cell Lines and Reagents

Human glioblastoma U87-MG (ATCC HTB-14) and microglia HMC3 (ATCC CRL-3304) cell lines were cultured in DMEM High Glucose medium (Sigma–Aldrich S.r.l., Milan, Italy) supplemented with 10% fetal bovine serum (FBS), 50 IU/mL penicillin, and 100 µg/mL streptomycin (Sigma–Aldrich S.r.l.) at 37 °C in a humidified atmosphere with 5% CO_2_; the medium was renewed three times per week.

The peptide Aβ_25–35_ (Sigma Aldrich S.r.l., Milan, Italy) was dissolved in sterile deionized water at a concentration of 1 mM and stocked in various aliquots at −20 °C. Aggregates were prepared with aliquots incubated at 37 °C for four days prior to treatment.

#### 4.2.2. Cell Viability Assay

Cells were seeded in 96-well plates at a density of 7 × 10^4^ cells/well and cultured for 24 h. The cells were treated with Aβ_25–35_ and/or compounds at the indicated concentrations for specified times. After the treatment, cell viability was measured by MTT assay (88417-1G, Sigma–Aldrich S.r.l., Milan, Italy). Briefly, 10 µL of the MTT solution (5 mg/mL) was added to each well and incubated for 4 h at 37 °C. Then all but 25 µL of supernatant were removed and 75 µL of DMSO was added into each well. The absorbance was measured at 570 nm with a microplate reader (Microplate Reader, Model 680, Bio-Rad, Milano, Italy).

#### 4.2.3. Analysis of Interleukin Release After Inflammatory Stimulus

The concentrations of the pro-inflammatory IL-6 and the anti-inflammatory IL-10 were analyzed by specific ELISA assays (RAB0306 and RAB0244, Sigma-Aldrich, Milan, Italy) on the collected culture media. Microglial cells were treated with selected concentrations of test compounds for 30 min and then stimulated for 24 h with appropriate inflammatory stimuli: LPS (10 µg/mL) and TNF (50 ng/mL). When the TAAR1 antagonist (EPPTB, 1 µM) was administered, it was added 15 min before the agonist. After 24 h, the media were collected and stored at −20 °C until the analysis.

#### 4.2.4. Gene Expression Analysis

The transcriptional expression levels of autophagy genes were explored by real-time PCR in both U87MG cells and *C. elegans*.

Concerning RNA extraction from cells, U87MG cells were cultured at a density of 3 × 105 cells/well in a 6-well plate in a final volume of 1.5 mL/well. Twenty-four hours after seeding, the cells were treated with Aβ_25–35_ and SG2 and its analogues. Total RNA was extracted using the Direct-zol RNA MiniPrep (Zymo Research, Irvine, CA, USA) including the on-column DNase I treatment, following the manufacturer’s instructions.

In contrast, total RNA extraction from whole worms was performed using chloroform extraction with TRIzol (Thermo Fisher Scientific, Milan, Italy). Briefly, samples resuspended in TRIzol were incubated at room temperature for 10 min and centrifuged at 14,000× *g* for 10 min at 4 °C. The supernatant was transferred into a fresh RNAse free Eppendorf tube and 200 µL of chloroform were added. After 15 s of vortex and 3 min of incubation at room temperature, samples were centrifuged at 12,000× *g* for 15 min at 4 °C. The obtained top layers (clear) were transferred into a new RNAse free Eppendorf tube and 500 µL of isopropanol were added. After 10 min of incubation at room temperature, centrifugation at 12,000× *g* at 4 °C was performed. Obtained pellets were washed with 100 µL of 75% ethanol and centrifuged at 7500× *g* for 5 min at 4 °C. Supernatants were removed and the pellet dissolved in 25 µL RNAse free water.

After the retro-transcription step, performed by iScriptTM gDNA Clear cDNA Synthesis Kit (Bio-Rad, Milano, Italy), the relative quantities of cDNA samples were analyzed by real-time PCR experiments using SsoAdvanced Universal SYBR Green Supermix on CFX-Connect real-time PCR detection system instrument (Bio-Rad, Milano, Italy). The PCR cycle programme consisted of an initial 30 s denaturation at 95 °C followed by 40 cycles of 5 s denaturation at 95 °C and 15 s annealing/extension at 60 °C. A final melting protocol with ramping from 65 °C to 95 °C with 0.5 °C increments of 5 s was performed for verification of amplicon specificity and primer dimer formation. Negative control of retro-transcription was performed to exclude any interference from residual genomic DNA contamination. Sequences of the primers for real-time PCR are reported in Table S1 (see Supplementary Materials).

#### 4.2.5. Statistical Analysis

All data are reported as mean ± SEM. Statistical analysis was performed by Student’s t test or one-way analysis of variance (ANOVA), followed by Dunnett’s and Tukey’s multiple comparison tests. The threshold of statistical significance was set at *p* < 0.05. Data analysis was performed by GraphPad Prism 8.4 statistical programme (GraphPad Software Inc., San Diego, CA, USA).

### 4.3. ADME-Tox Profiling

The ADME-tox profiling was assessed in vitro. The evaluation was performed at 10 µM in triplicate. Briefly, the cytotoxicity was tested at 24 and 48 h in osteosarcoma (U2OS), human breast adenocarcinoma, (MCF7) and human embryonic kidney, (HEK293) cell lines and in lung fibroblast (hTERT). The hERG-related cardiotoxicity was evaluated by means of the hERG fluorescence polarization assay; the cytochrome (Cyp)450 inhibition was assessed against the following isoforms CYP1A2, CYP2C9, CYP2C19, CYP2D6, and CYP3A4. Finally, the off-target liability was evaluated by fluorimetric assays using HDAC isoforms HDAC4, HDAC6, HDAC8, HDAC9), SIRT7, PDE4C1 and Aurora B kinase.

This panel of assays was performed applying the protocols already described by us [20].

### 4.4. C. elegans Experiments

#### 4.4.1. Media

Standard conditions were used for the propagation of *C. elegans*. The Nematode Growth Medium NGM (1 mM CaCl_2_, 1 mM MgSO_4_, 5 μg/mL cholesterol, 25 mM KH_2_PO_4_ pH 6, 15.75 g/L agar, 2.7 g/L NaCl, 5.75 g/L Peptone) culturing plates were prepared by filling sterile 9 cm Petri dishes with 20 mL of sterile hot NGM and left to cool to room temperature. *E. coli* strain OP50 cultures were obtained by inoculating 250 mL of LB broth (Peptone 10 g/L, NaCl 10 g/L, yeast extract 5 g/L) with OP50 and incubating for 16 h at 37 °C to afford a cloudy, saturated OP50 solution. The solution was brought to 10% of the original volume and 350 μL were added to each of the previously prepared plates and allowed to dry completely.

To obtain animals of uniform age, the nematodes were synchronized by bleaching them with a 3:2 mixture of 3.5% bleach and 4 N NaOH and subsequently placing the recovered eggs, after several washings with water, in multi-well plates filled with M9 buffer (3 g/L KH_2_PO_4_, 6 g/L Na_2_HPO_4_, 5 g/L NaCl, 1 μM MgSO_4_) for 16 h at 20 °C. After hatching, newborn L1 worms were counted, transferred into NGM plates seeded with OP50 and kept at 20 °C for 24 h (day 2) until they reached L4 stage. On day 3 after synchronization, adult worms were recovered and transferred into bacteria seeded plates with NGM and 5-fluorodeoxyuridine (FUdR) (75 μM per plate unless stated otherwise) to stop worm reproduction and maintain the same number of animals throughout the experiment. These plates were incubated at 25 °C to induce Aβ_1–42_ precipitation and subsequently paralysis.

#### 4.4.2. Strains

The following strains were used in this study: Bristol N2, wildtype (wt); GMC101, dvIs100 [unc-54p:: Aβ_1–42_::unc-54 30-UTR + mtl-2p::GFP]. The gene unc-54p:: Aβ_1–42_ expresses the full length human Aβ_1–42_ peptide which self-aggregates in vivo in body wall muscle cells.

#### 4.4.3. Compound Administration

To perform the experiment, various groups of bacteria-seeded FUdR NGM plates were prepared based on the number of compounds to be tested. Solutions of the compounds at the relative concentrations were prepared in 1% DMSO in water and 2.2 mL of each solution was added to the designated treated GMC101 plates in order to enrich the culture medium with the compound. The plates were then left to dry under a sterile vertical laminar flow hood. The same procedure was followed for the control GMC101 and N2 designated plates, to which only a plain 1% DMSO solution was added.

#### 4.4.4. *C. elegans* Experimental Protocol

All *C. elegans* populations were maintained at 20 °C and were synchronized by bleaching to recover the eggs, which have been hatched for 16 h at 20 °C. The next day, L1 larvae were transferred to standard bacteria-seeded NGM plates for 48 h to reach L4 stage. At L4 stage worms were transferred to FUdR NGM plates and left at 25 °C for 3 days (D1, D2 and D3 of adulthood) to induce paralysis. On D4 of adulthood the animals were transferred again into FUdR NGM plates containing the compound or the control solution as previously explained. Approximately 1000 worms were placed in each plate. Data was collected on D5, D6 and D7 of adulthood using the WF-NTP platform.

#### 4.4.5. Motility Assay with WF-NTP

At different ages (D5, D6 and D7) the motility, expressed in Bends Per Minute (BPM), of the worm populations was monitored via the WF-NTP platform. To perform data collection an adequate amount (approx. 500 worms) of individuals were transferred on unseeded NGM motility plates and suspended in 5 mL of M9 buffer. The motility plates were loaded on the platform and, after a 30 s wait to avoid stress-related behaviour, a 1 min video was recorded at 20 fps. A custom-made tracking code was used for video analysis. Up to 500 worms were tracked in each video, unless stated otherwise.

#### 4.4.6. Statistical Analysis

Statistical analyses were performed using GraphPad Prism version 6.0 for Windows (GraphPad Software, San Diego, CA, USA). Data were subjected to one-way analysis of variance for mean comparison. Data are reported as mean ± SEM. Differences at *p* < 0.05 were considered statistically significant, and the different *p* values are reported as *p* ≤ 0.05, ** *p* ≤ 0.01, *** *p* ≤ 0.001, or **** *p* ≤ 0.0001.

### 4.5. UHPLC-HR-MS Measurement of SG2, SG22 and SG23 Uptake by C. elegans

#### 4.5.1. Sample Preparation

Briefly, GMC101 AD worms were cultured as explained above and prepared for the experiment following the protocol of Yuan et al. [43]. The worms were washed from NGM plates with M9 buffer and counted with the WF-NTP software (https://www.rstudio.com, June 2024). Subsequently the same worm batches were transferred in a 15 mL Falcon tube. Next 50 µL of 1 mM DMSO solution of the compound were added to the tube. Finally, the suspension of worms was brought to a volume of 5 mL with M9 to reach a compound concentration of 10 µM in the suspension. A control suspension was also prepared with AD worms treated with 50 µL of pure DMSO. A number of worms between 200 and 500 units was used in each experiment. All the experiments were run in triplicate.

The suspensions were left incubating in agitation at room temperature for 4 h. After incubation, the suspensions were centrifuged at 3200 rpm for 3 min and the supernatant was decanted. The worm pellets treated with tested compounds were then washed consecutively with 10 mL of PBS + 1% BSA, followed by 10 mL of PBS + 0.01% Tween20, and finally 10 mL of PBS. After the final wash, the supernatant was decanted and the worm pellets were stored at −80 °C. The frozen pellets were then sonicated for 15 min in an ice bath with 1 mL of MeOH/H_2_O/HCl (50:49.9:0.1, *v*/*v*/*v*) solution, and then frozen again. This sonication-freezing process was repeated a total of three times to ensure complete rupturing of the worms. At the end of this cycle, the suspension was centrifuged at 3200 rpm for 3 min and the supernatant was collected, evaporated and stored at −20 °C for analysis.

#### 4.5.2. UHPLC-HR-MS Analysis of *C. elegans*

Analyses were conducted with a Vanquish Flex Binary UHPLC coupled to a high-resolution Q Exactive Plus Orbitrap-based FT-MS (HR-Orbitrap/MS) mass spectrometer equipped with an electrospray ionization (ESI) source (Thermo Fisher Scientific Inc., Bremen, Germany). The LC column used was a Kinetex Biphenyl column (100 × 2.1 mm, 2.6 µm) provided by a security GuardTM Ultra Cartridge (Phenomenex, Bologna, Italy) maintained at a temperature of 30 °C. The mobile phase consisted of a mixture of 0.1% *v*/*v* formic acid in water (solvent A) and 0.1% *v*/*v* formic acid in methanol (solvent B) with a linear gradient increasing from 5 to 100% solvent B over 25 min splitting system 1:1 to DAD and MS detector. Dry extract was dissolved in methanol solution (UHPLC-grade from Deltek, Pozzuoli, Italy) at a concentration of 1.0 mg/mL, and 10 µL of the sample were injected into an LC-MS system and eluted at a flow rate of 0.3 mL/min. ESI-HR mass spectra were recorded in positive ESI mode using a scan range *m*/*z* 200–500. UV spectra were recorded at 290 nm. Xcalibur 4.1 software (Thermo Fisher Scientific Inc., Bremen, Germany) was used for data elaboration.

#### 4.5.3. UHPLC-MS-DAD Analyses of *C. elegans* Media

##### Sample Preparation

The original stock solution of SG-22 (1 mg/mL in DMSO) was stored at −20 °C and brought to room temperature prior to use. Standard solutions of the analyte were prepared by diluting original stock solutions with ACN. Acetanilide was used as internal standard (IS). A solvent based standard calibration curve was constructed by using neat solutions obtained by dilution with ACN of the standard solutions, in the way of obtaining a range of concentration of 0.1–1 µg/mL (ppm) containing the IS at 0.44 µg/mL (ppm).

The untreated blank sample consisted of a solution of compound SG-22 1% DMSO (5 mL total volume) in Buffer M9 of concentration 10 µM. To this, 10 mL of PBS + 1% BSA solution, 10 mL of PBS + 0.01% TWEEN 20 and 10 mL of PBS are then added in order. Extraction was performed as reported: in a 5 mL Eppendorf, 1 mL of supernatant of untreated blank sample solution and 1 mL of ethyl acetate were shaken with vortex for 10 s at 25 Hz. After the two phases were separated, 400 µL of ethyl acetate is taken and frozen. The extracted untreated blank solution of SG-22 by AcOEt (500 µL) was dried and then dissolved in 480 µL of ACN and 20 µL of the internal standard solution. The recovery of the compound in the worm environment was measured by means of calibration curves and is reported in Table S3.

##### Analysis

UPLC-MS-DAD analyses were performed on an Acquity UPLC Waters instrument coupled with an Acquity QDa Waters mass spectrometer (probe temperature: 600 °C; ESI capillary voltage 1.5 kV; cone voltage 15 V; mass range 60–1000 Da) and a PDA eλ Detector (wavelength range 200–800 nm). Quantitation was performed using Selected Ion Monitoring (SIM) in positive ion mode at the following *m*/*z* values: 341 for SG-22, 136 for IS. The analytes were separated on an Acquity UPLC 2.1 × 100 mm column, BEH C18, 1.7 μm, using 95/5 H_2_O/ACN (A) and 5/95 H_2_O/ACN (B) as the mobile phases at a flow rate of 0.5 mL/min. Separation was achieved under the following chromatographic conditions: 0% B for 1.5 min then increased to 100% B in 6.5 min and maintained for 1 min. The temperature of the chromatographic column was maintained at 40 °C. Injection volume was 0.25 µL. Each measurement was performed in triplicate. The obtained calibration curves had a great linearity, with a R^2^ ≥ 0.99 in the 1–200 ng/mL range. No compound carryover was encountered between the sample injections.

## 5. Conclusions

In conclusion, this work reinforces the concept that thyronamine-like analogues represent a versatile and promising scaffold for multi-target intervention in AD, capable of simultaneously modulating autophagy, neuroinflammation, and cellular stress responses. Among the compounds tested, SG-23 emerges as a particularly attractive candidate for further preclinical development, combining efficacy with an improved safety profile.

## Data Availability

The original contributions presented in this study are included in the article/Supplementary Material. Further inquiries can be directed to the corresponding authors.

## Supplementary Materials

The following supporting information can be downloaded at: https://www.mdpi.com/article/10.3390/molecules31162833/s1.

## References

[1] Twiss E., McPherson C., Weaver D.F. (2025). Global Diseases Deserve Global Solutions: Alzheimer’s Disease. Neurol. Int..

[2] Nichols E., Steinmetz J.D., Vollset S.E., Fukutaki K., Chalek J., Abd-Allah F., Abdoli A., Abualhasan A., Abu-Gharbieh E., Akram T.T. (2022). Estimation of the Global Prevalence of Dementia in 2019 and Forecasted Prevalence in 2050: An Analysis for the Global Burden of Disease Study 2019. Lancet Public Health.

[3] Menzies F.M., Fleming A., Caricasole A., Bento C.F., Andrews S.P., Ashkenazi A., Füllgrabe J., Jackson A., Jimenez Sanchez M., Karabiyik C. (2017). Autophagy and Neurodegeneration: Pathogenic Mechanisms and Therapeutic Opportunities. Neuron.

[4] Yin F. (2023). Lipid Metabolism and Alzheimer’s Disease: Clinical Evidence, Mechanistic Link and Therapeutic Promise. FEBS J..

[5] Cai Y., Liu J., Wang B., Sun M., Yang H. (2022). Microglia in the Neuroinflammatory Pathogenesis of Alzheimer’s Disease and Related Therapeutic Targets. Front. Immunol..

[6] Barmaki H., Nourazarian A., Khaki-Khatibi F. (2023). Proteostasis and Neurodegeneration: A Closer Look at Autophagy in Alzheimer’s Disease. Front. Aging Neurosci..

[7] Breijyeh Z., Karaman R. (2020). Comprehensive Review on Alzheimer’s Disease: Causes and Treatment. Molecules.

[8] Fox N.C., Belder C., Ballard C., Kales H.C., Mummery C., Caramelli P., Ciccarelli O., Frederiksen K.S., Gomez-Isla T., Ismail Z. (2025). Treatment for Alzheimer’s Disease. Lancet.

[9] Bellusci L., Laurino A., Sabatini M., Sestito S., Lenzi P., Raimondi L., Rapposelli S., Biagioni F., Fornai F., Salvetti A. (2017). New Insights into the Potential Roles of 3-Iodothyronamine (T1AM) and Newly Developed Thyronamine-Like TAAR1 Agonists in Neuroprotection. Front. Pharmacol..

[10] Rogowski M., Bellusci L., Sabatini M., Rapposelli S., Rahman S.M., Chiellini G., Assadi-Porter F.M. (2019). Lipolytic Effects of 3-Iodothyronamine (T1AM) and a Novel Thyronamine-Like Analog SG-2 through the AMPK Pathway. Int. J. Mol. Sci..

[11] Runfola M., Perni M., Yang X., Marchese M., Bacci A., Mero S., Santorelli F.M., Polini B., Chiellini G., Giuliani D. (2021). Identification of a Thyroid Hormone Derivative as a Pleiotropic Agent for the Treatment of Alzheimer’s Disease. Pharmaceuticals.

[12] Bellusci L., Runfola M., Carnicelli V., Sestito S., Fulceri F., Santucci F., Lenzi P., Fornai F., Rapposelli S., Origlia N. (2020). Endogenous 3-Iodothyronamine (T1AM) and Synthetic Thyronamine-like Analog SG-2 Act as Novel Pleiotropic Neuroprotective Agents Through the Modulation of SIRT6. Molecules.

[13] Chiellini G., Nesi G., Sestito S., Chiarugi S., Runfola M., Espinoza S., Sabatini M., Bellusci L., Laurino A., Cichero E. (2016). Hit-to-Lead Optimization of Mouse Trace Amine Associated Receptor 1 (mTAAR1) Agonists with a Diphenylmethane-Scaffold: Design, Synthesis, and Biological Study. J. Med. Chem..

[14] Kouznetsov V.V. (2024). Exploring Acetaminophen Prodrugs and Hybrids: A Review. RSC Adv..

[15] Simplício A.L., Clancy J.M., Gilmer J.F. (2008). Prodrugs for Amines. Molecules.

[16] Millucci L., Ghezzi L., Bernardini G., Santucci A. (2010). Conformations and Biological Activities of Amyloid Beta Peptide 25-35. Curr. Protein Pept. Sci..

[17] Polini B., Ricardi C., Bertolini A., Carnicelli V., Rutigliano G., Saponaro F., Zucchi R., Chiellini G. (2023). T1AM/TAAR1 System Reduces Inflammatory Response and β-Amyloid Toxicity in Human Microglial HMC3 Cell Line. Int. J. Mol. Sci..

[18] Alvarez J., Alvarez-Illera P., Santo-Domingo J., Fonteriz R.I., Montero M. (2022). Modeling Alzheimer’s Disease in Caenorhabditis Elegans. Biomedicines.

[19] Runfola M., Sestito S., Bellusci L., La Pietra V., D’Amore V.M., Kowalik M.A., Chiellini G., Gul S., Perra A., Columbano A. (2020). Design, Synthesis and Biological Evaluation of Novel TRβ Selective Agonists Sustained by ADME-Toxicity Analysis. Eur. J. Med. Chem..

[20] Runfola M., Sestito S., Gul S., Chiellini G., Rapposelli S. (2020). Collecting Data through High Throughput in Vitro Early Toxicity and Off-Target Liability Assays to Rapidly Identify Limitations of Novel Thyromimetics. Data Brief.

[21] Fang E.F., Hou Y., Palikaras K., Adriaanse B.A., Kerr J.S., Yang B., Lautrup S., Hasan-Olive M.M., Caponio D., Dan X. (2019). Mitophagy Inhibits Amyloid-β and Tau Pathology and Reverses Cognitive Deficits in Models of Alzheimer’s Disease. Nat. Neurosci..

[22] Kabeya Y., Mizushima N., Ueno T., Yamamoto A., Kirisako T., Noda T., Kominami E., Ohsumi Y., Yoshimori T. (2000). LC3, a Mammalian Homologue of Yeast Apg8p, Is Localized in Autophagosome Membranes after Processing. EMBO J..

[23] Zhang Z., Yang X., Song Y.-Q., Tu J. (2021). Autophagy in Alzheimer’s Disease Pathogenesis: Therapeutic Potential and Future Perspectives. Ageing Res. Rev..

[24] Baeken M.W., Bekbulat F., Körschgen H., Clement A.M., Behl C. (2025). The Sigma-1 Receptor as a Neurohomeostatic Decision Hub for GABARAP-Mediated Receptor Trafficking and Macroautophagy. Front. Mol. Biosci..

[25] Saxton R.A., Sabatini D.M. (2017). mTOR Signaling in Growth, Metabolism, and Disease. Cell.

[26] Kumar S., Lombard D.B. (2018). Functions of the Sirtuin Deacylase SIRT5 in Normal Physiology and Pathobiology. Crit. Rev. Biochem. Mol. Biol..

[27] Gao C., Jiang J., Tan Y., Chen S. (2023). Microglia in Neurodegenerative Diseases: Mechanism and Potential Therapeutic Targets. Signal Transduct. Target. Ther..

[28] Bradaia A., Trube G., Stalder H., Norcross R.D., Ozmen L., Wettstein J.G., Pinard A., Buchy D., Gassmann M., Hoener M.C. (2009). The Selective Antagonist EPPTB Reveals TAAR1-Mediated Regulatory Mechanisms in Dopaminergic Neurons of the Mesolimbic System. Proc. Natl. Acad. Sci. USA.

[29] Rutigliano G., Bandini L., Sestito S., Chiellini G. (2020). 3-Iodothyronamine and Derivatives: New Allies Against Metabolic Syndrome?. Int. J. Mol. Sci..

[30] Krishna S., Borrel A., Huang R., Zhao J., Xia M., Kleinstreuer N. (2022). High-Throughput Chemical Screening and Structure-Based Models to Predict hERG Inhibition. Biology.

[31] Iacopetta D., Ceramella J., Catalano A., Scali E., Scumaci D., Pellegrino M., Aquaro S., Saturnino C., Sinicropi M.S. (2023). Impact of Cytochrome P450 Enzymes on the Phase I Metabolism of Drugs. Appl. Sci..

[32] Zhang Y., Wang Z., Wang Y., Jin W., Zhang Z., Jin L., Qian J., Zheng L. (2024). CYP3A4 and CYP3A5: The Crucial Roles in Clinical Drug Metabolism and the Significant Implications of Genetic Polymorphisms. PeerJ.

[33] Perni M., Challa P.K., Kirkegaard J.B., Limbocker R., Koopman M., Hardenberg M.C., Sormanni P., Müller T., Saar K.L., Roode L.W.Y. (2018). Massively Parallel *C. elegans* Tracking Provides Multi-Dimensional Fingerprints for Phenotypic Discovery. J. Neurosci. Methods.

[34] Koopman M., Peter Q., Seinstra R.I., Perni M., Vendruscolo M., Dobson C.M., Knowles T.P.J., Nollen E.A.A. (2020). Assessing Motor-Related Phenotypes of Caenorhabditis Elegans with the Wide Field-of-View Nematode Tracking Platform. Nat. Protoc..

[35] Klionsky D.J., Abeliovich H., Agostinis P., Agrawal D.K., Aliev G., Askew D.S., Baba M., Baehrecke E.H., Bahr B.A., Ballabio A. (2008). Guidelines for the Use and Interpretation of Assays for Monitoring Autophagy in Higher Eukaryotes. Autophagy.

[36] Palmisano N.J., Meléndez A. (2016). Detection of Autophagy in Caenorhabditis Elegans Using GFP::LGG-1 as an Autophagy Marker. Cold Spring Harb. Protoc..

[37] Wu J., Geng C., Liu L., Tang Y. (2025). Advancement of Disease-Modifying Therapy of Alzheimer’s Disease: From the Perspective of New Revised Criteria for Diagnosis and Staging of Alzheimer’s Disease. Med. Plus.

[38] Naseer A., Mir S.S., Takacs-Vellai K., Nazir A. (2021). Sirtuins and Autophagy in Age-Associated Neurodegenerative Diseases: Lessons from the *C. elegans* Model. Int. J. Mol. Sci..

[39] Hein Z.M., Vishnumukkala T., Karikalan B., Alkatiri A., Hussan F., Jagadeesan S., Kamaruzzaman M.A., Che Ramli M.D., Che Mohd Nassir C.M.N., Gopalakrishna P.K. (2025). Autophagy and Alzheimer’s Disease: Mechanisms and Impact Beyond the Brain. Cells.

[40] Lagu B., Senaiar R.S., Kluge A.F., Mallesh B., Ramakrishna M., Bhat R., Patane M.A. (2020). Addressing hERG Activity While Maintaining Favorable Potency, Selectivity and Pharmacokinetic Properties of PPARδ Modulators. Bioorg. Med. Chem. Lett..

[41] Chisholm A.D., Xu S. (2012). The Caenorhabditis elegans epidermis as a model skin. II: Differentiation and physiological roles. Wiley Interdiscip. Rev. Dev. Biol..

[42] Sundaram M.V., Pujol N. (2024). The Caenorhabditis elegans cuticle and precuticle: A model for studying dynamic apical extracellular matrices in vivo. Genetics.

[43] Yuan T., Ma H., Liu W., Niesen D.B., Shah N., Crews R., Rose K.N., Vattem D.A., Seeram N.P. (2016). Pomegranate’s Neuroprotective Effects against Alzheimer’s Disease Are Mediated by Urolithins, Its Ellagitannin-Gut Microbial Derived Metabolites. ACS Chem. Neurosci..

