# Supplementary material for: Novel Insights into the Pleiotropic Neuroprotective Action of Synthetic Halogen Free Thyronamine-like Analogues
Source: Molecules. 2026 Aug 14;31(16):2833. doi: 10.3390/molecules31162833 (PMC13515986; doi:10.3390/molecules31162833)
Supplement: Supplementary file 1 [file molecules-31-02833-s001.zip › molecules-4437301-supplementary.pdf]

SUPPLEMENTARY MATERIAL

Novel Insights into the Pleiotropic Neuroprotective Action of Synthetic Halogen Free Thyronamine-like Analogues

Massimiliano Runfola [1,†], Beatrice Polini [2,†], Anna Mazzierli [2], Lorenzo Raffellini [1], Fabio Di Ricco [1], Italo Cirone [1], Simona Sagona [1], Sheraz Gul [3], Marco Lessi [4], Angela Rosa Cuzzola [4], Rosarita D'Orsi [4], Fabio Bellina [4], Clementina Manera [1,5], Grazia Chiellini [2,5,*] and Simona Rapposelli [1,5,*]

1 Department of Pharmacy, University of Pisa, Via Bonanno 6, 56126 Pisa, Italy; massimiliano.runfola@unipd.it (M.R.); lorenzo.raffellini@phd.unipi.it (L.R.); fabio.diricco@phd.unipi.it (F.D.R.); italo.cirone@phd.unipi.it (I.C.); simona.sagona@unipi.it (S.S.); clementina.manera@unipi.it (C.M.)

2 Department of Surgical, Medical, Molecular Pathology and Critical Care, University of Pisa, Via Savi 10, 56100 Pisa, Italy; beatrice.polini@unipi.it (B.P.); anna.mazzierli@phd.unipi.it (A.M.)

3 Fraunhofer Institute for Translational Medicine and Pharmacology ITMP, Discovery Research ScreeningPort, 22525 Hamburg, Germany; sheraz.gul@itmp.fraunhofer.de

4 Department of Chemistry and Industrial Chemistry, University of Pisa, Via Giuseppe Moruzzi 13, 56124 Pisa, Italy; marco.lessi@unipi.it (M.L.); angela.rosa.cuzzola@unipi.it (A.R.C.); rosarita.dorsi@unipi.it (R.D.); fabio.bellina@unipi.it (F.B.)

5 Center for Instrument Sharing, University of Pisa (CISUP), Lungarno Pacinotti 43, 56126 Pisa, Italy

* Correspondence: grazia.chiellini@unipi.it (G.C.); simona.rapposelli@unipi.it (S.R.)

† These authors contributed equally to this work.

SUPPLEMENTARY MATERIAL

Supplementary info index.

Synthesis. S3
Characterisation. S3
Figure S1 .... S7
Table S1. Primer sequences used for gene expression analysis S7
ADME-Tox profiling. S9
***C. elegans* motility histograms** S9
Quantification of compounds (SG-*2, SG-22, and SG-23) uptake in C. elegans.* S10
SG-22 mass spec spectra S12
UHPLC-MS-DAD analysis of liquid media S14

SUPPLEMENTARY MATERIAL

Synthesis

In **scheme S1** is reported the synthesis of compounds SG-2, SG-22, and SG-23 [Chiellini et al J Med. Chem 2016 **2016**, *59*, 9825–9836].

Scheme S1

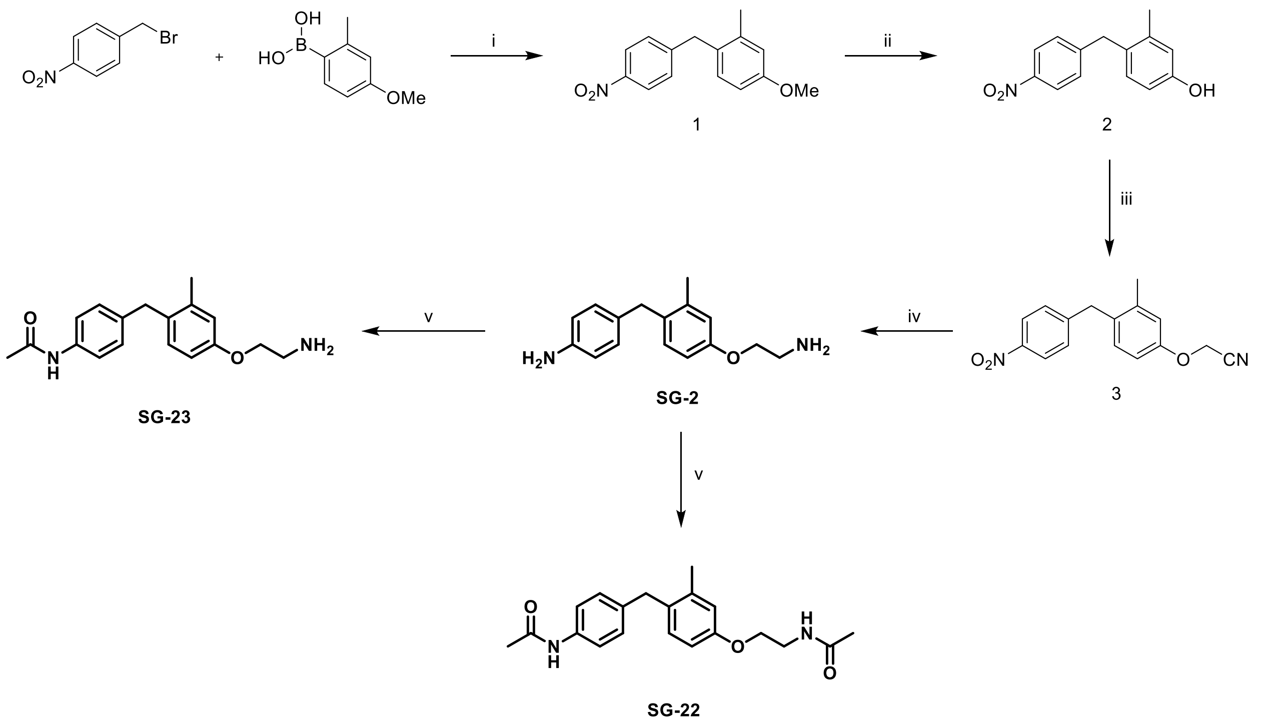

Reagents and conditions. (i) K_2CO_3, Pd(dppf)Cl_2, acetone, H_2O, 130 °C (MW), 30 min; (ii) BBr_3, DCM, -10 °C, 2 h; (iii) $BrCH_2CN$, Cs_2CO_3, DMF, rt, 30 h; (iv) $LiAlH_4$, $AlCl_3$, THF, reflux, 12 h; (v) Ac_2O/$NaHCO_3$, rt, 1 h.

Characterisation

Here are reported the ^{1}H-NMR and ^{13}C-NMR spectra of **SG-2**, **SG-22** and **SG-23**.

SUPPLEMENTARY MATERIAL

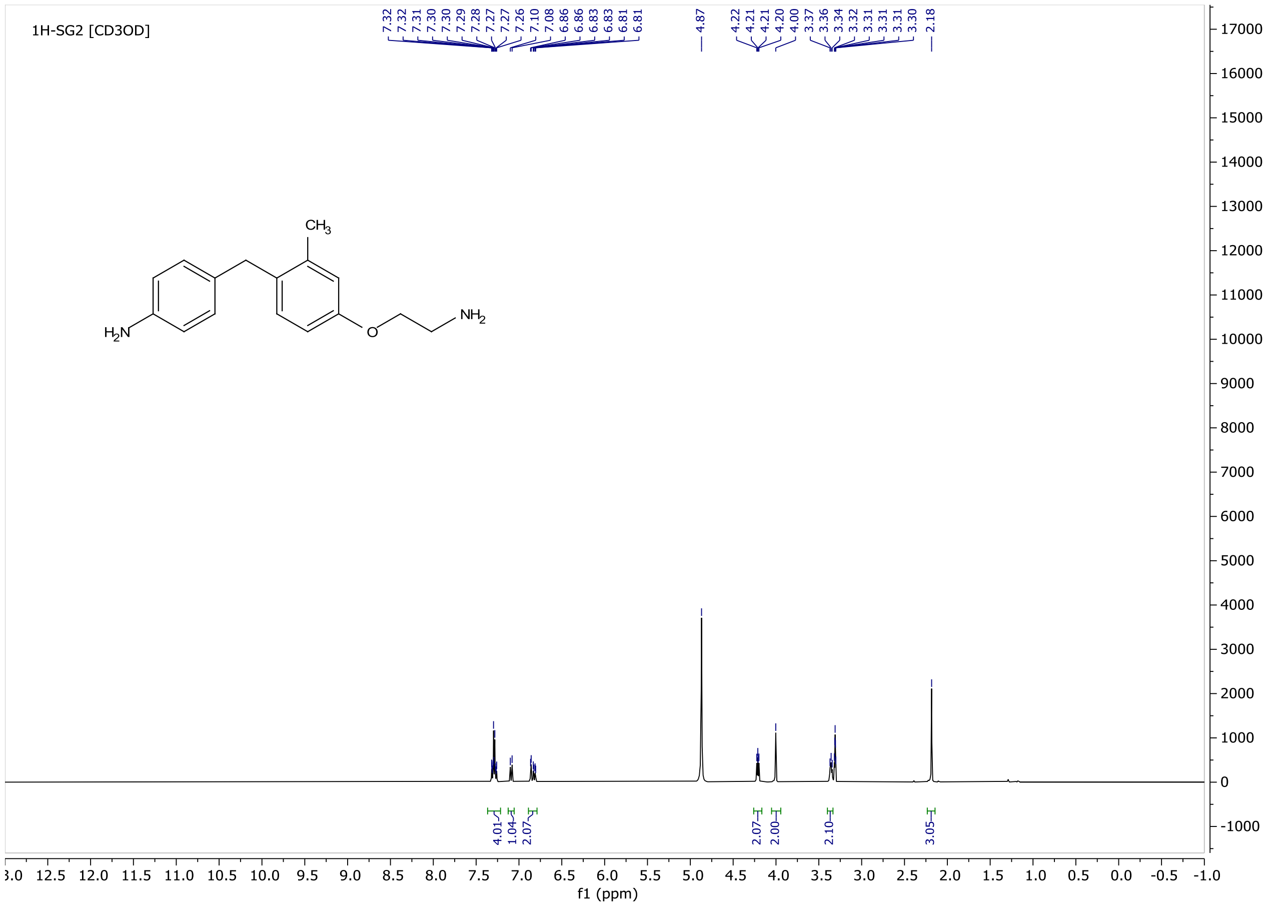

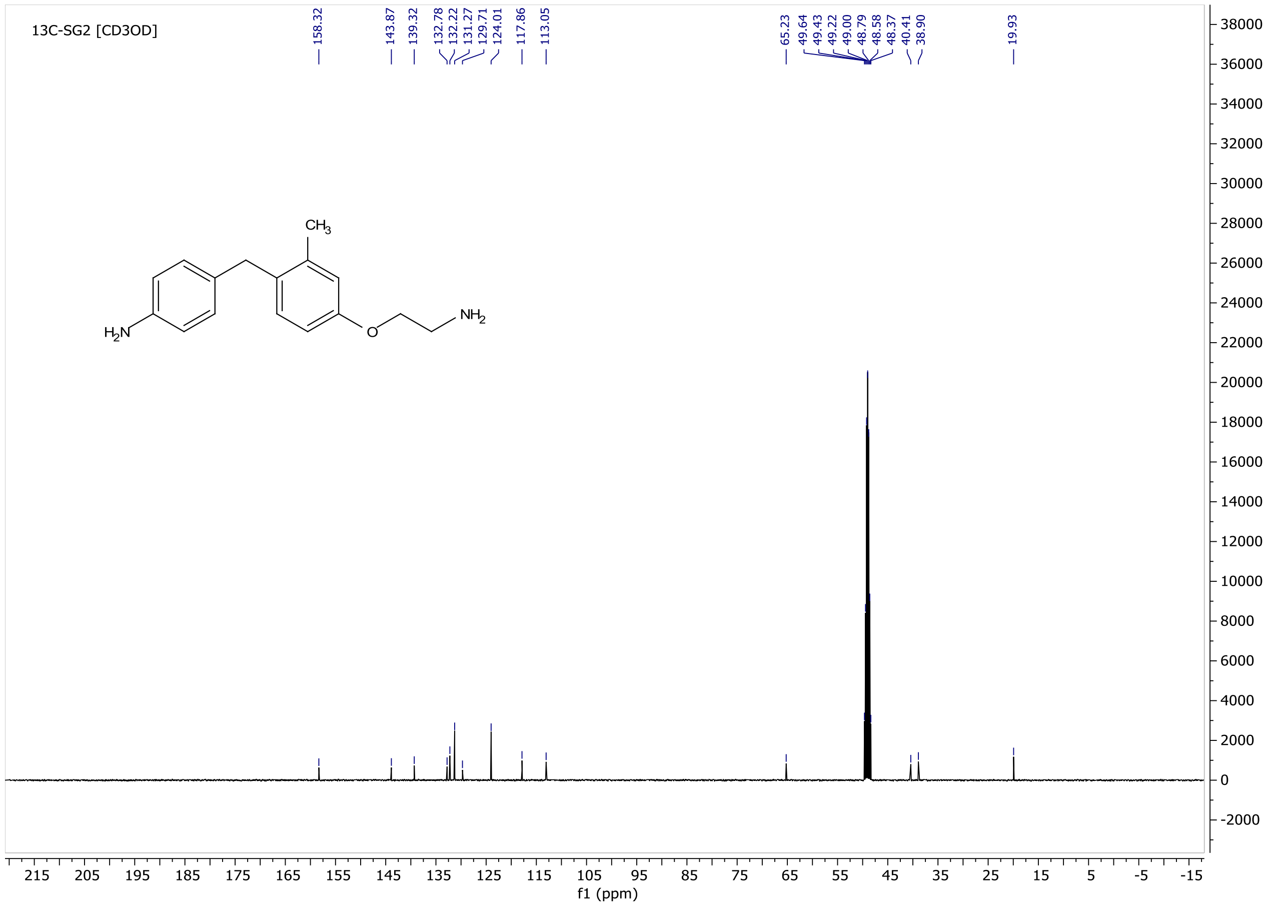

SUPPLEMENTARY MATERIAL

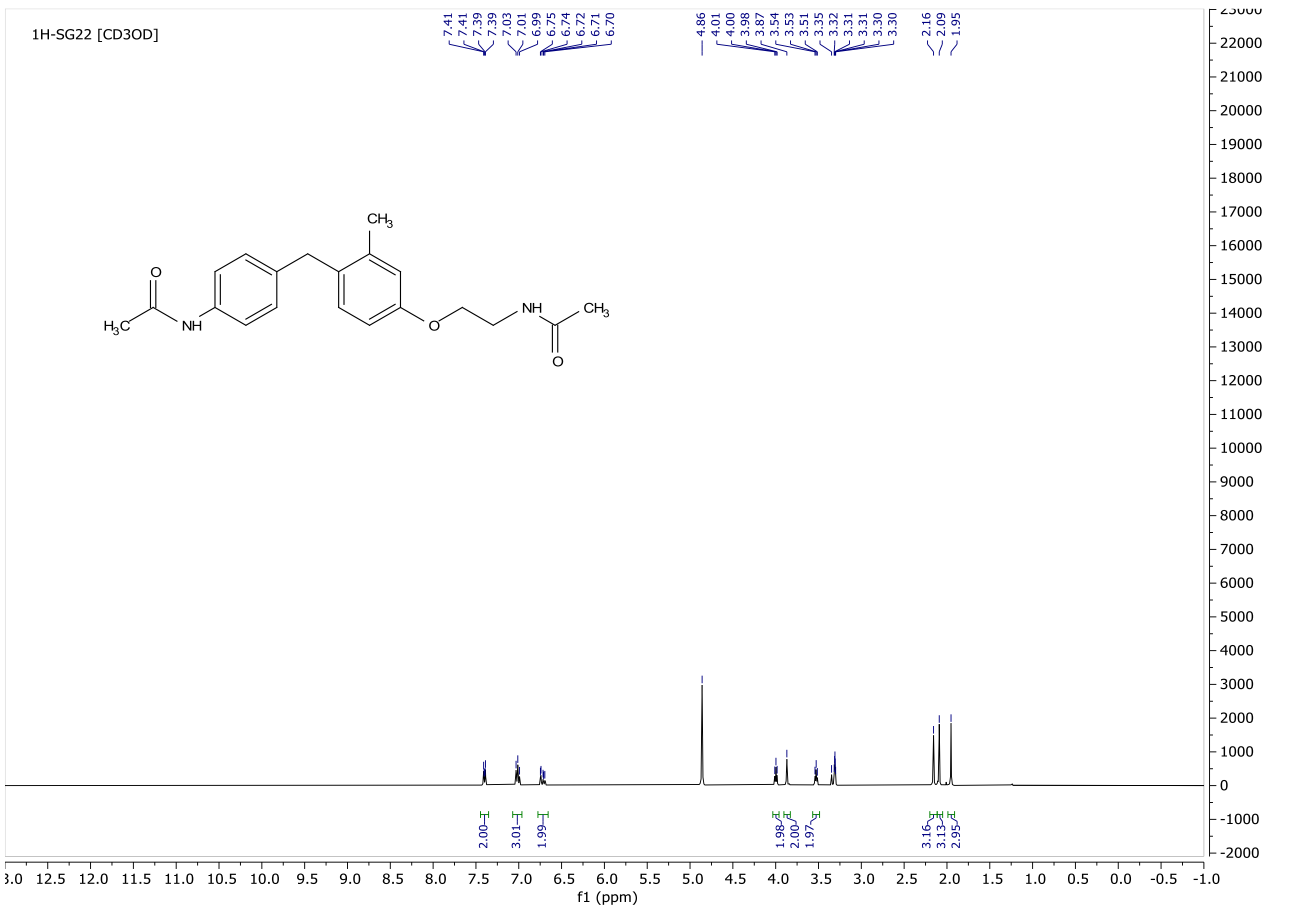

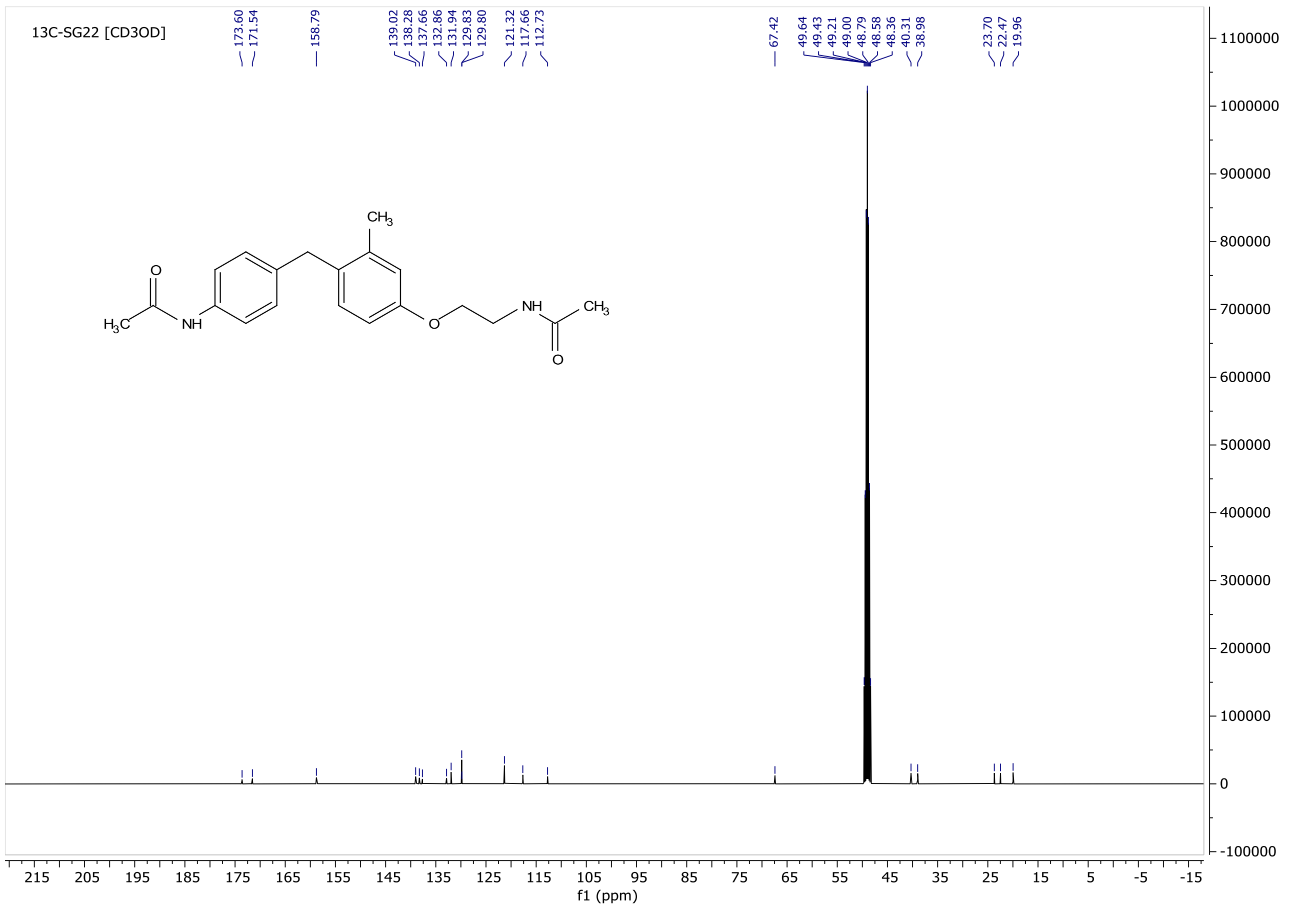

SUPPLEMENTARY MATERIAL

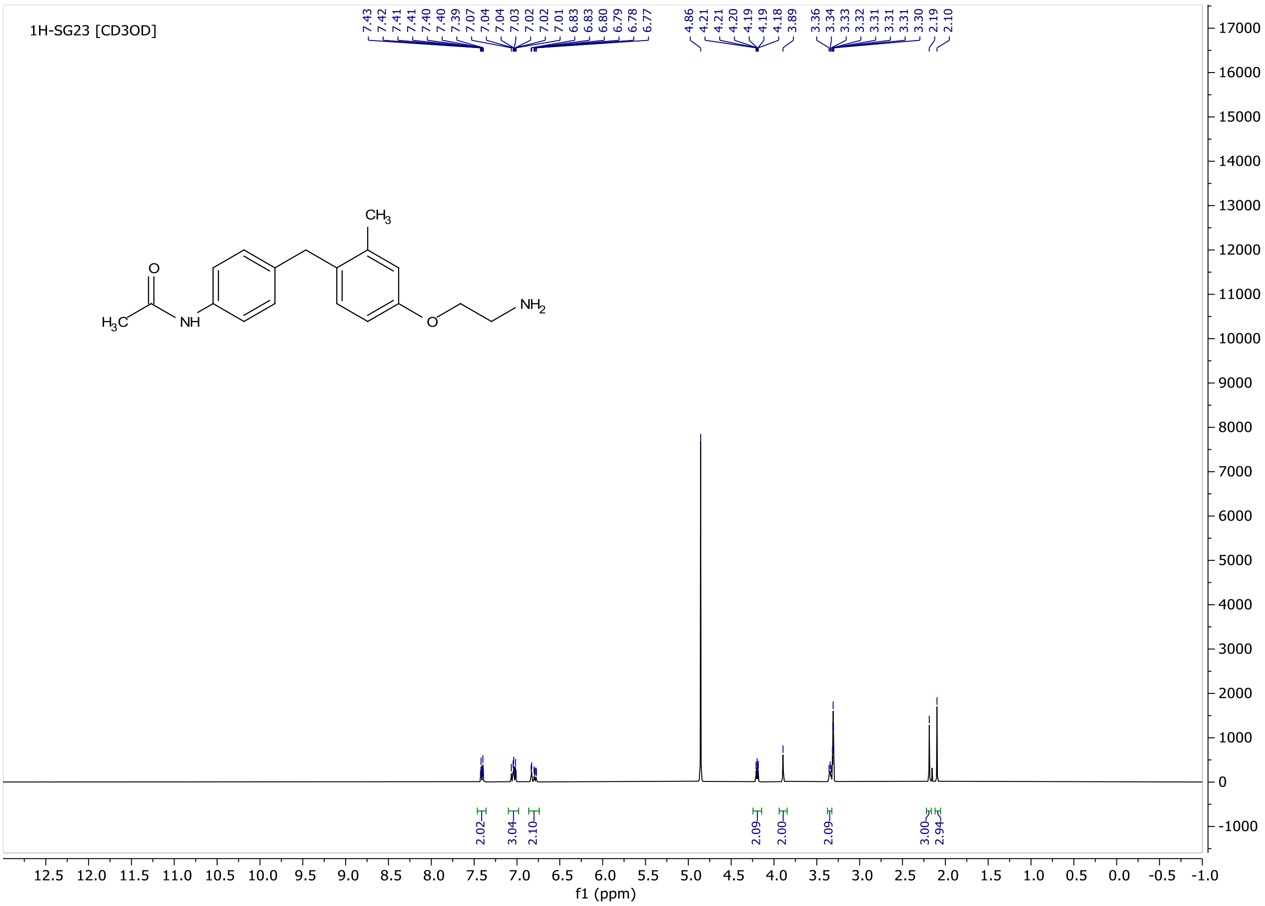

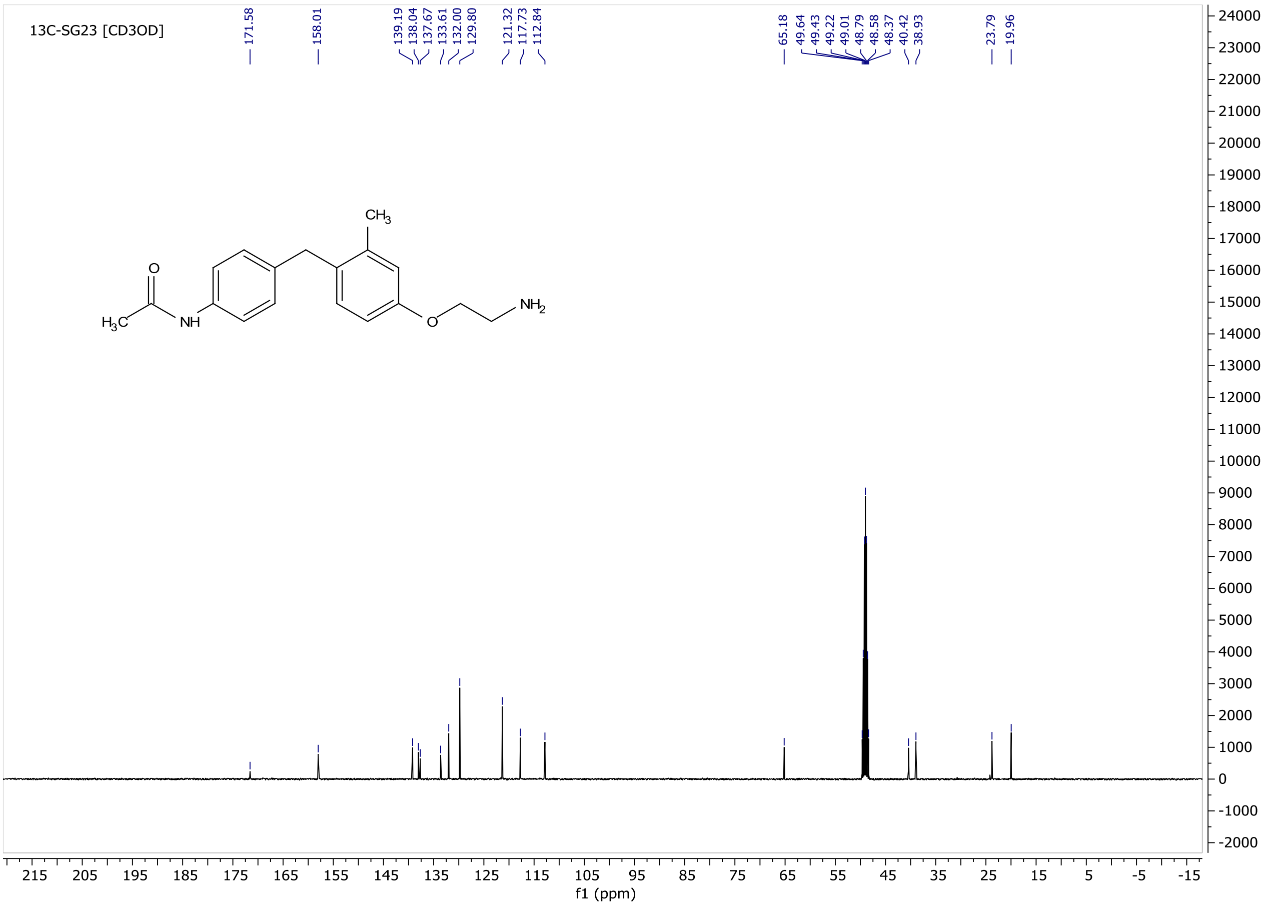

SUPPLEMENTARY MATERIAL

Figure S1. Evaluation of the cytotoxicity of the three tested compounds in HMC3 microglial cells. Cell viability was assessed by the MTT assay following treatment with each compound at concentrations of 0.1, 1, and 10 µM. None of the compounds significantly affected cell viability at any of the tested concentrations, confirming that the experimental conditions used throughout the study were non-cytotoxic. Data are presented as mean ± SEM of three independent experiments, each performed in technical quadruplicate.

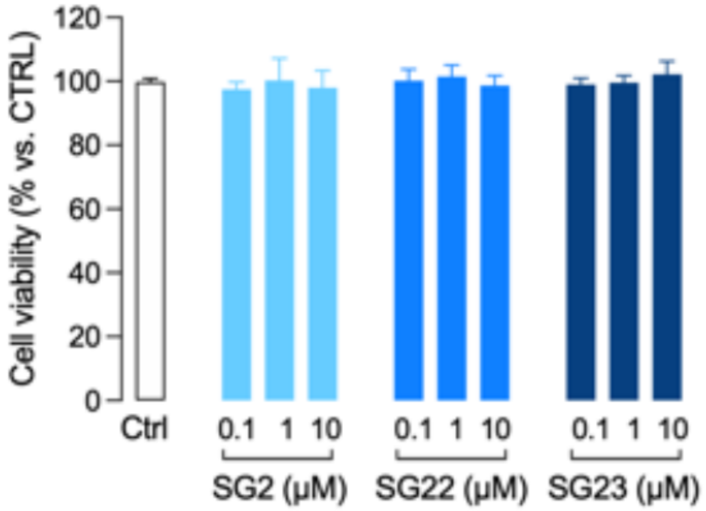

Table S1. Primer sequences used for gene expression analysis.

Forward and reverse primer sequences used for qPCR analysis of human and *C. elegans* genes. The prefix "h" indicates human genes, while "ce" refers to C. elegans orthologs. Accession numbers are reported for each gene.

GENE	ACCESSION NUMBER	PRIMERS	
hLC3B	NM_022818	*forward*	5' AGTCTTCTCTTCAGGTTCAC 3'
		reverse	5' CTCACACAGCCCGTTTAC 3'
hMTOR	NM_004958	*forward*	5' TGCCTTCACAGATACCCAGTA 3'
		reverse	5' AGACCTCACAGCCACAGA 3'
hSIRT1	NM_012238	*forward*	5'GTAGGCGGCTTGATGGTAAT 3'
		reverse	5' GGGTTCTTCTAAACTTGGACTCT 3'
hSIRT5	NM_012241	*forward*	5' AATAACTAAAGCCCGCCTCAA 3'
		reverse	5' CAAATCTGGTTTCGTGTGGAC 3'
hSIRT6	NM_016539	*forward*	5' CTCCTCCGCTTCCTGGTC 3'

		reverse	5’ TTACACTTGGCACATTCTTCC 3’
hGAPDH	NM_002046	*forward*	5’ CCCTTCATTGACCTCAACTACATG 3’
		reverse	5’ TGGGATTTCCATTGATGACAAGC 3’
ceBEC1	NM_068443.4	forward	5’ CTGATTCACTGGTTGACAATGGT 3’
		reverse	5’ CTGATTCACTGGTTGACAATGGT 3’
ceLGG1	NM_062876.8	*forward*	5’ ACCCAGACCGTATTCCAGTG 3’
		reverse	5’ ACGAAGTTGGATGCGTTTTC 3’
ceLET-363	NM_0591486	*forward*	5’ CGGTGACAGACATCCATCCA 3’
		reverse	5’ TCAACGCTATGGAAGTAACTGGAT 3’
ceSQST1	NM_001268679.3	*forward*	5’ CAGCCCTACCATCAGTGAGC 3’
		reverse	5’ CTGAACTTCCTCGACAACAGG 3’
ceATG5	NM_182058.5	*forward*	5’TGGCAATACAGAAAGTGCTCAG 3’
		reverse	5’ TACGAACATCCCAGCCGC 3’
ceSIR-2.1	NM_001268555.5	*forward*	5’ GAAGATGACGATGATACACGGAAT 3’
		reverse	5’ CCAGGAAAGACGGTTTGATGAG 3’
ceACTIN1	NM_073418.9	*forward*	5’ ACGACGAGTCCGGCCCATCC 3’
		reverse	5’ GAAAGCTGGTGGTGACGATGGTT 3’
ceAMA1	NM_068122.9	*forward*	5’ TGGAACTCTGGAGTCACACC 3’
		reverse	5’ CATCCTCCTTCATTGAACGG 3’

ADME-Tox profiling

SG-2, **SG-22**, and **SG-23** were evaluated for their ADME-Tox properties. The panel of assays included cytotoxicity on U2OS, HEK293 and hTERT cell lines; hERG channel inhibition; epigenetic modulation (SIRT7, HDAC4, HDAC6, HDAC8, HDAC9); off-target liability on PDE4C1 and Aurora B; metabolic stability (CYP1A2, CYP2C9, CYP2D6, CYP3A4).

Assay	***SG-2***			***SG-22***			***SG-23***			
	AVG	*SD*	*n*	*AVG*	*SD*	*n*	*AVG*	*SD*	*n*	*Assay Z'*
U2OS 24h	**-7.9**	*8.5*	*3*	**20.7**	*13.1*	*3*	**28.2**	*13.4*	*3*	*0.59*
HEK293 24h	**12.7**	*2.3*	*3*	**2.9**	*1.4*	*3*	**11.1**	*1.8*	*3*	*0.75*
hTERT 24h	**18.9**	*2.7*	*3*	**1.1**	*2.9*	*3*	**13.7**	*3.3*	*3*	*0.73*
U2OS 48h	**11.7**	*2.4*	*6*	**-2.7**	*4.2*	*6*	**14.6**	*4*	*6*	*0.79*
HEK293 48h	**17**	*2.7*	*3*	**13.4**	*6*	*3*	**13.5**	*1.7*	*3*	*0.78*
hTERT 48h	**16.6**	*1.6*	*3*	**10.9**	*2.1*	*3*	**7.2**	*4.3*	*3*	*0.74*
hERG	**83.9**	*9.7*	*3*	**-8.2**	*10.2*	*3*	**43.5**	*10.1*	*3*	*0.43*
SIRT 7	**22.3**	*4.3*	*3*	**16.1**	*5.4*	*3*	**9.6**	*0.4*	*3*	*0.72*
HDAC4	**17.8**	*3*	*3*	**16.4**	*1.3*	*3*	**15.8**	*1.1*	*3*	*0.83*
HDAC6	**-32.1**	*6.5*	*3*	**-23.6**	*3*	*3*	**-16.1**	*16.5*	*3*	*0.85*
HDAC8	**4.8**	*1.6*	*3*	**-1.1**	*1.9*	*3*	**13**	*3.8*	*3*	*0.71*
HDAC9	**16.7**	*4.2*	*3*	**8**	*3.8*	*3*	**12.4**	*1.1*	*3*	*0.89*
PDE4C1	**6.4**	*4.4*	*3*	**20.4**	*2.9*	*3*	**17.1**	*20.6*	*3*	*0.62*
Aurora B	**14.1**	*5*	*3*	**4.4**	*2*	*3*	**10**	*1.3*	*3*	*0.81*
CYP1A2	**33.3**	*36.5*	*3*	**51.9**	*2*	*3*	**35.3**	*38*	*3*	*0.83*
CYP2C9	**98**	*0.1*	*3*	**35**	*2.9*	*3*	**94.8**	*0.2*	*3*	*0.76*
CYP2C19	**98.2**	*0.6*	*3*	**45.3**	*2.7*	*3*	**97.3**	*0.4*	*3*	*0.83*
CYP2D6	**73.7**	*4*	*3*	**31.2**	*6.2*	*3*	**48**	*11*	*3*	*0.78*
CYP3A4	**86.2**	*1.3*	*2*	**42**	*1.1*	*2*	**48.7**	*1.2*	*3*	*0.77*

Table S2. ADME-Tox profiling data from Figure 1. Percentage of inhibition or cytotoxicity for each assay are reported as the average (AVG) from replicate (n) measurements together with the standard deviation (SD). The Z′ is also reported for each assay.

C. elegans motility histograms.

SG-2, **SG-22**, and **SG-23** were tested at 10 µM at D4 of adulthood. Readouts to gain motility data as bends per minute (BPM) were acquired at D5, D6 and D7.

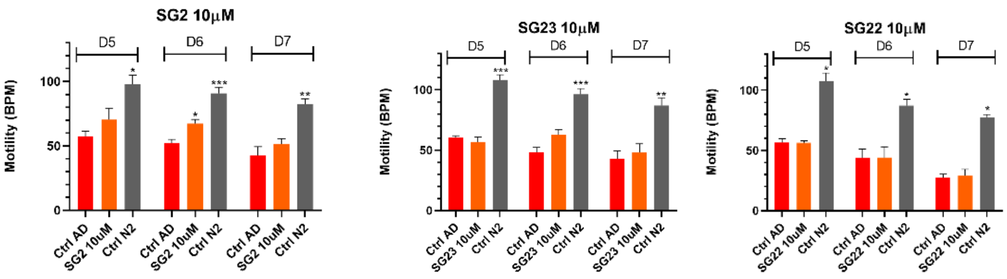

Figure S2. Nematode motility histograms. Compounds **SG-2**, **SG-22**, **SG-23** were tested at 10 μM in triplicate. Motility data were acquired at D5, D6 and D7. Data are expressed as mean ± SEM. The statistical comparison was performed using Ordinary one-way ANOVA. * p ≤ 0.05, ** p ≤ 0.01, *** p ≤ 0.001 (vs AD control). Ctrl AD: untreated GMC101 worms (negative control); Ctrl N2: wildtype worms used as positive control. BPM: Bends per minute.

Quantification of compounds (SG-*2, SG-22, and SG-23)* uptake in *C. elegans*.

Calibration curve of standard sample solutions of SG-2 SG-22 and **SG-23.** The curve were used to determine the concentration of sample recovered by nematode extraction as reported in the manuscript

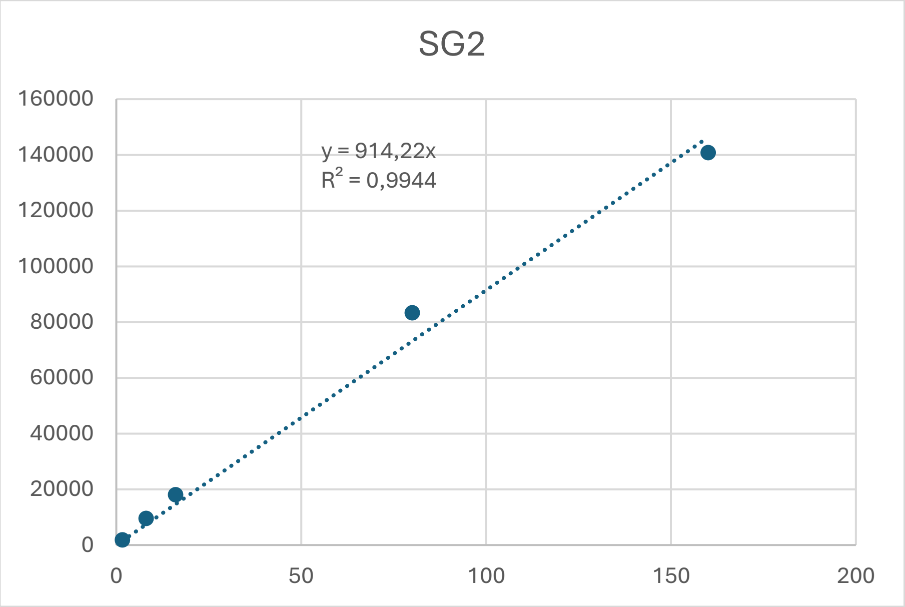

LOD 0.8 ng/mL; LOQ 2.5 ng/mL

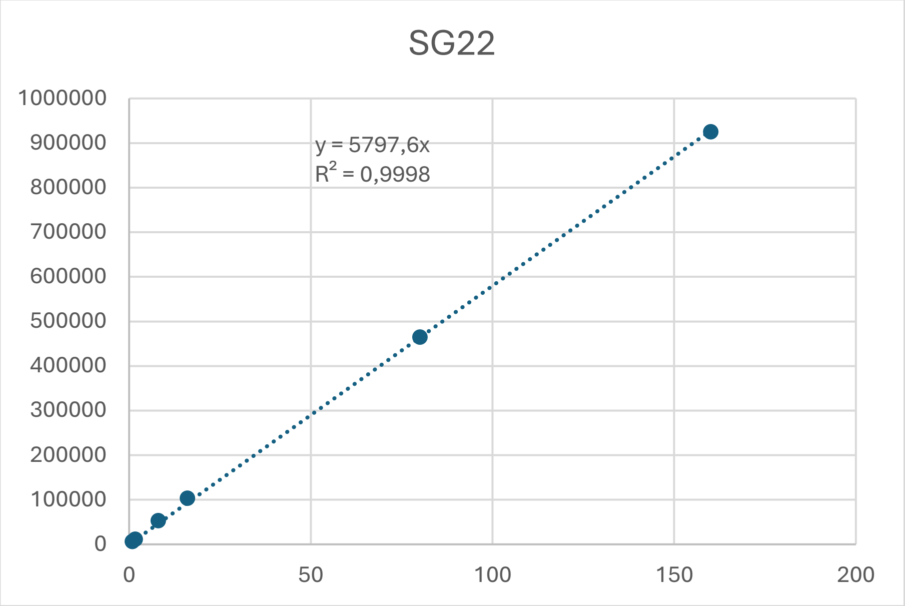

LOD 0.15 ng/mL; LOQ 0.5 ng/mL

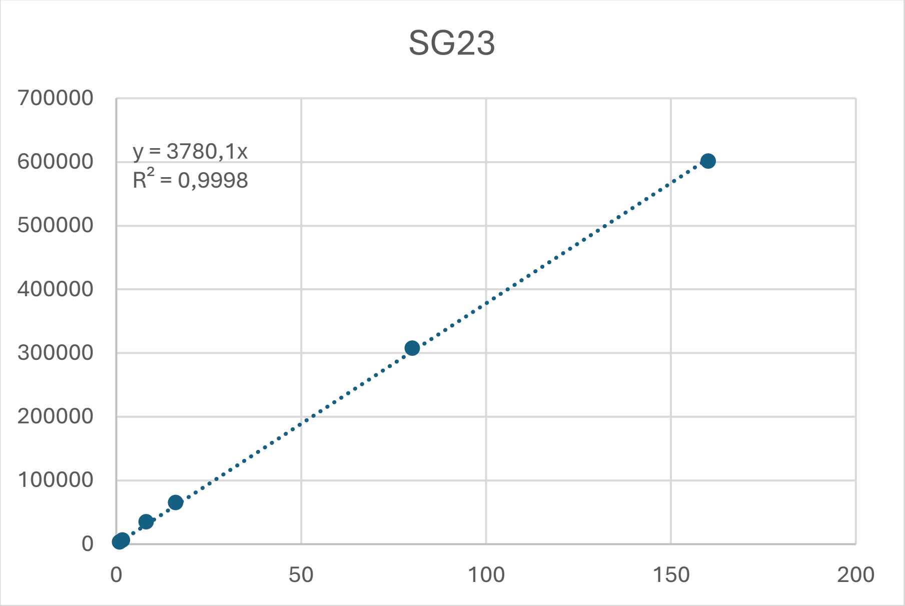

LOD 0.15 ng/mL; LOQ 0.5 ng/mL

Figure S3.

Worm pellets were processed for compound extraction, and uptake was quantified by UPLC-MS analysis of the resulting extraction supernatant. Reported concentrations (ng/mL) refer to 1 mL of supernatant obtained after worm lysis and extraction. The total amount of each compound was normalized to the number of worms per sample and expressed as ng per worm (table S2).

Table S3.

Treatment	Number of worms	Compound	Total amount exctracted (ng/mL)	Normalized uptake (ng/worm)
**SG2**	526	**SG2**	7.2	0.0137
**SG22**	807	**SG22**	2,7	0.00335
		**SG23**	1,6	0.00198
**SG23**	144	**SG23**	10,6	0.0736

SG-22 mass spec spectra. Graphs represent the unknown peaks obtained through MS experiments on **SG-22** treated nematodes, namely (356, 342 and 328).

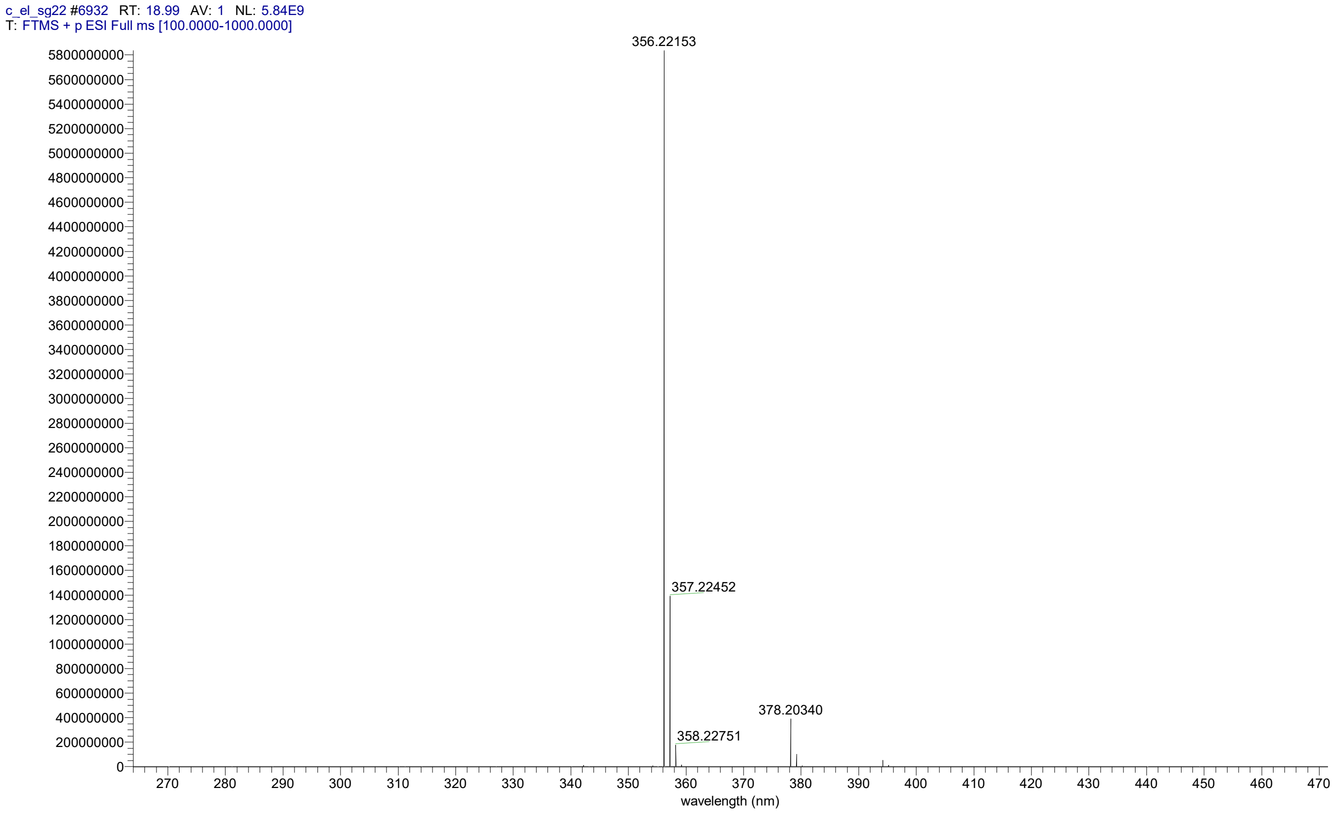

SUPPLEMENTARY MATERIAL

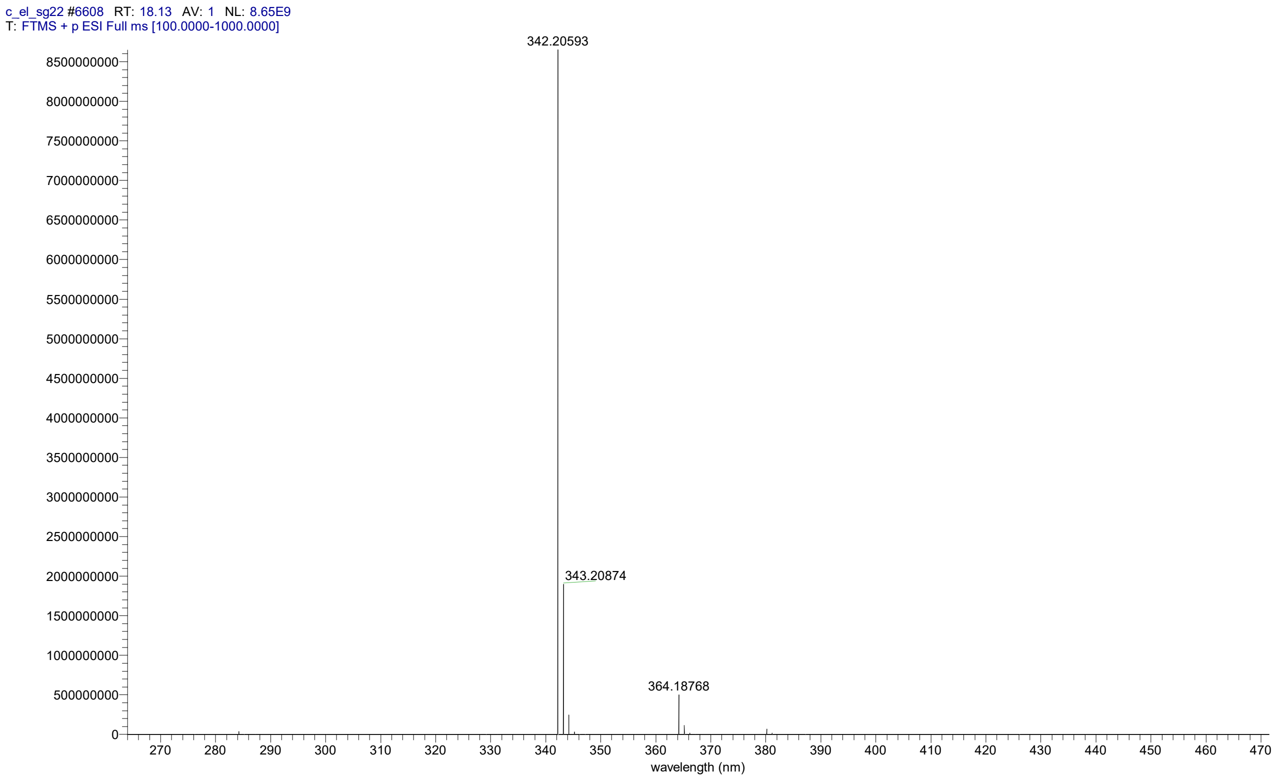

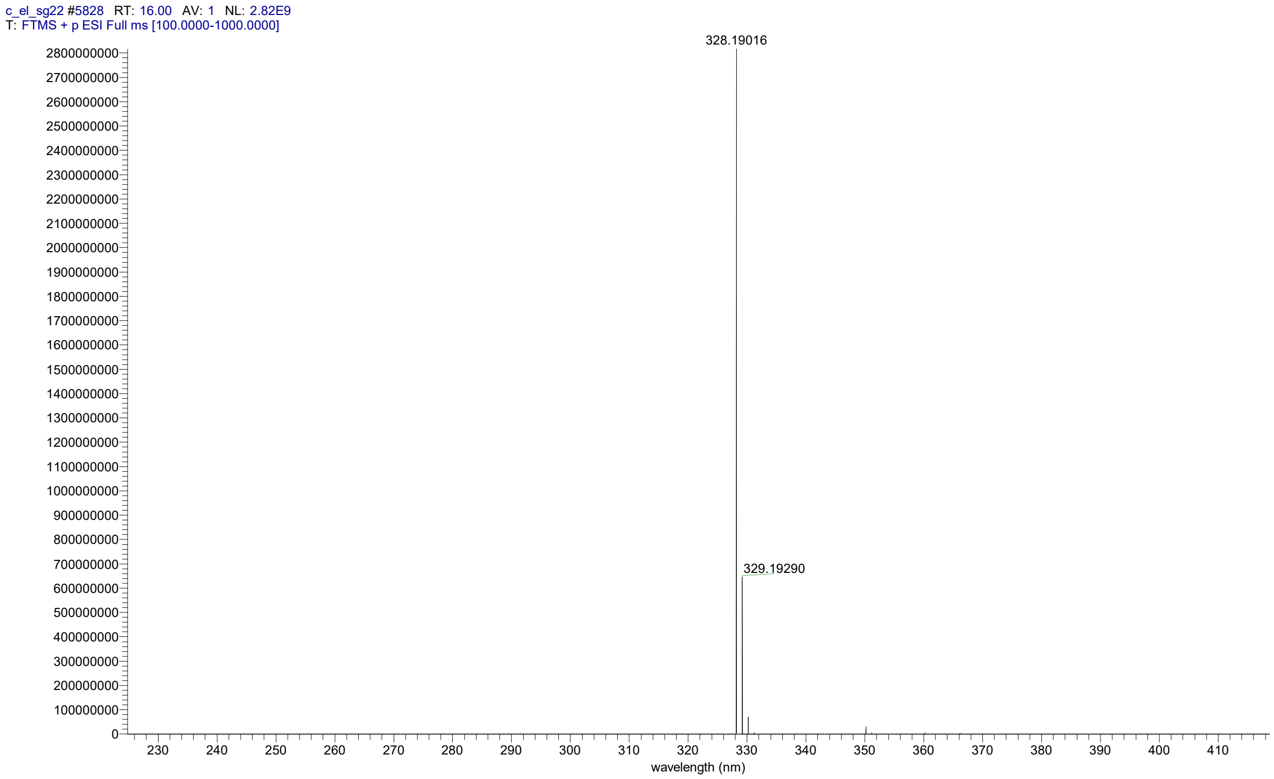

UHPLC-MS-DAD analysis of liquid media

Calibration curve of standard sample solution (SG-22)

Calibration curve obtained by a standard solution of SG-22 with the protocol reported in the paper. The curve was used to determine the concentration of sample recovered by the untreated blank sample solution and the treated sample solution.

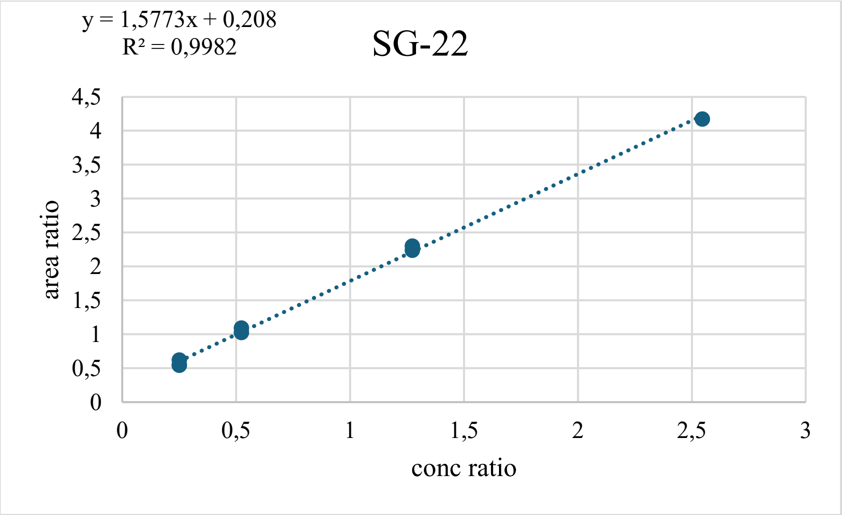

Figure S4. Calibration curve of SG22 standard solution using acetanilide as standard.

First, the untreated blank solutions were extracted with different solvents or firstly lyophilised and the extracted with solvents, in order to obtain the maximum possible recovery. Recovery with different extraction solvents is reported in Table S3 together with the recovery of SG22-treated sample. The recovery of the compound in the worm environment was measured by means of calibration curve.

The extracted untreated blank solution of SG22 by AcOEt (500 ml) was dried and then dissolved in 480 ul of ACN and 20 ul of the internal standard solution.

<u>Untreated blank sample lyophilised and extracted procedure</u>

Entry 2: 1 mL of the supernatant solution was lyophilised, extracted twice with 0.5 mL of ACN for a total of 1 mL. Centrifuged to deposit salts analysed in the presence of the internal standard as described above.
Entry 3: to the solution of entry A 1% of DMSO was added and stirred for 1h, then centrifuged and analysed in the presence of internal standard.
Entry 4: 1 mL of the supernatant solution was lyophilised, extracted twice with 1 mL of DCM and centrifuged. The solvent was evaporated and the residue dissolved in 1 mL of ACN and analysed in the presence of the internal standard as described above.
Entry 5: 1 mL of the supernatant solution was lyophilised, extracted twice with 1 mL of MeOH and centrifuged. The solvent was evaporated and the residue dissolved in 1 mL of ACN and analysed in the presence of the internal standard as described above

Entry	Sample	Extraction solvent	Recovery
**1**	Blank sample	AcOEt	**91.7% ± 0.7**
**2**	Blank sample lyophilised	ACN	33.9% ± 0.3
**3**	Blank sample lyophilised	ACN+1%DMSO	54.6% ± 0.4
**4**	Blank sample lyophilised	DCM	13.1% ± 0.1
**5**	Blank sample lyophilised	MeOH	24.2% ± 0.2
**6**	SG22-treated sample	AcOEt	**90.9% ± 0.7**

Table S3. In entry **1** the recovery of sample was from direct extraction of untreated blank supernatant solution with AcOEt. Each measurement was performed in triplicate. In entry **2-5** the recovery of the sample SG-22 from the extraction of lyophilised SG-22 supernatant solution with different solvents. Each measurement was performed in triplicate.

Results showed that direct blank sample extraction with AcOEt is the best method to quantify the sample in the worm environment. The same procedure was applied to quantify the amount of tested compound remained in medium (SG22-trated sample), and a recovery of 90.9 ± 0.7 % was measured. This data compared with the entry 1 of the **Table S3**, in which the same compound in untreated blank solution was recovered in 91.7 ± 0.7 % confirm the non-absorption of **SG-22** by nematodes
